# Co(II)-Catalyzed Picolinamide-Directed C(sp^3^)-S Bond Formation with *N*-(phenylsulfanyl)succinimides

**DOI:** 10.3390/molecules30224462

**Published:** 2025-11-19

**Authors:** Jinjing Qin, Shaodong Zhou, Jinwen Luo, Guodong Wang, Kai Wang

**Affiliations:** 1 College of Chemical and Biological Engineering, Zhejiang University, Hangzhou 310058, China; 2 Zhejiang Anglikang Pharmaceutical Co., Ltd., Shengzhou 312400, China; 3 School of Pharmacy, Qilu Medical University, Zibo 255300, China; 4 College of Medical Engineering, Jining Medical University, Jining 272002, China

**Keywords:** Co(II)-catalyzed, C(sp^3^)-S bond formation, green strategies

## Abstract

Herein, we disclose a novel and efficient cobalt-catalyzed cross-coupling strategy for picolinamide-directed direct C(sp^3^)-H bond formation with *N*-(phenylsulfanyl)succinimides. This method enables the direct construction of C(sp^3^)–S bonds under mild conditions and exhibits excellent functional group tolerance along with a broad substrate scope. Notably, the catalytic system achieves oxidative C–H functionalization without relying on costly or environmentally detrimental oxidants, offering a more sustainable and practical alternative for C–S bond formation.

## 1. Introduction

Aromatic chalcogen-based frameworks, particularly aryl thioethers, constitute a class of highly valuable molecular scaffolds that are widely present in numerous biologically active natural products [1,2,3], pharmaceutical agents [4,5,6], and functional materials [7,8]. Owing to the critical importance of these organosulfur compounds, significant research efforts have been directed toward developing efficient synthetic routes [9,10,11]. Conventional strategies for constructing C-S bonds have largely relied on transition metal-catalyzed cross-coupling reactions between aryl halides and thiols. However, these methods often suffer from narrow substrate scope, prefunctionalization requirements, and byproduct formation, driving the development of innovative catalytic strategies that offer milder conditions, broader compatibility, and improved sustainability.

**Figure 1:**
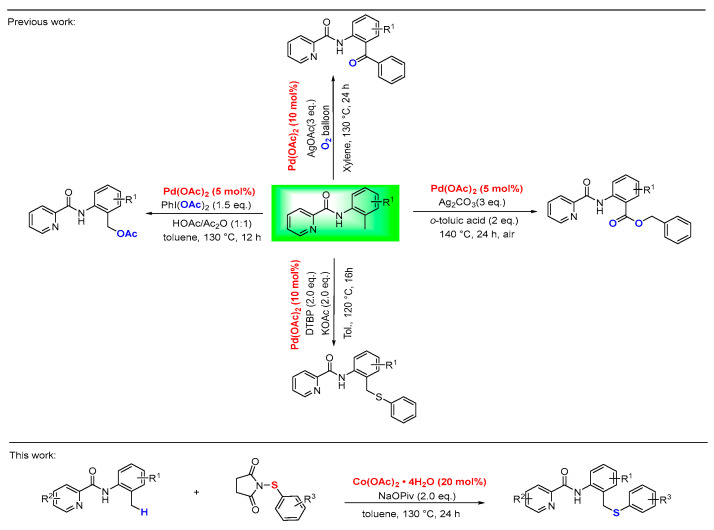
Picolinamide-directed functionalization of *γ*-C(sp^3^)-H bonds.

In recent years, with the participation of *N,N*-bidentate [12,13,14,15,16] coordination system, transition-metal-catalyzed C-H bond activation with a cyclic metallacycle intermediate has emerged as a useful pathway for novel and efficient strategy in the construction of C-X bonds (X = C, *N*, O, S). This approach eliminates circumventing the reliance on aryl halides required in traditional cross-coupling reactions, and significantly reduces halogenated byproducts, thereby improving atom economy and synthetic efficiency. Despite significant advances in C-H functionalization, research efforts have predominantly centered on C(sp^2^)-H bonds, whereas the functionalization of more inert C(sp^3^)-H bonds remains less explored [17,18,19,20]. Notably, palladium-catalyzed C(sp^3^)-H functionalization has been established as a robust strategy for constructing diverse organic molecules. For instance, in 2012, Zhang and colleagues reported a Pd-catalyzed arylation/oxidation of benzylic C-H bonds [21]. Subsequently, the same group achieved Pd-catalyzed acetoxylation of C(sp^3^)-H bonds to afford benzyl esters [22,23]. Related acetoxylation reactions were also documented by the research groups Huang [24]. In 2020, Xie and co-workers established the Pd-catalyzed direct benzylic C(sp^3^)-H chalcogenation with diaryl disulfides and diphenyl diselenide [25] (Figure 1). While significant progress has been made in picolinamide-directed C(sp^3^)-H functionalization, the field remains heavily reliant on expensive palladium catalysts, a reliance that substantially limits its economic viability and broader applicability. Research efforts are increasingly directed toward cobalt-catalyzed C-H functionalization, driven by the metal’s economic advantage and inherently sustainable profile compared to precious metal catalysts [26,27,28].

Herein, we report the first Co(II)-catalyzed, picolinamide-directed sulfurization of benzylic C(sp^3^)-H bonds, employing *N*-(phenylsulfanyl)succinimides as a source of sulfur ethers. This method exhibits excellent functional group tolerance and delivers diverse products in moderate to excellent yields.

## 2. Results and Discussion

We initiated our investigation using *N*-(*o*-tolyl)picolinamide (**1a**) and 1-(*p*-tolylthio)pyrrolidine-2,5-dione (**2a**) as model substrates, employing Co(OAc)_2_ as catalyst and KOAc as additive in toluene at 130 °C for 24 h. Satisfactorily, the desired product (**3a**) was obtained in 57% yield (Table 1, entry 1). Encouraged by this result, we screened various metal catalysts including CoBr_2_, Co(acac)_2_, NiCl_2_, CuBr_2_, Cu(OAc)_2_ and Fe(OAc)_2_. The comparative studies consistently identified Co(OAc)_2_ as the most effective catalyst for this specific transformation. In contrast, no reaction occurred in the absence of catalyst (Table 1, entries 2–8). Subsequently, a systematic examination on the impact of various additives was conducted, including NaOAc, K_2_CO_3_, Na_2_CO_3_ and NaOPiv, which were evaluated as alternatives to KOAc. Among them, NaOPiv proved superior to the others, yielding the target product in 72% (Table 1, entries 9–13). We also investigated other solvents, including *p*-xylene, DMSO, DMF and mesitylene. However, these solvent systems proved less favorable, leading to a concomitant decrease in yield to 58%, 43%, 48% and 61%, respectively (Table 1, entries 14–17). Finally, reducing the catalyst loading or changing the air atmosphere resulted in yield rates of 60, 13, and 76, respectively (Table 1, entris 18–20).

With the optimized reaction conditions established, we subsequently explored the substrate scope of sulfurization substrates (Table 2). The reaction demonstrated good functional group tolerance, accommodating a range of *N*-(phenylsulfanyl)succinimides bearing either electron-donating groups (EDGs) (**3a**, **3d**, **3g**) or electron-withdrawing groups (**3c**, **3e**, **3f**, **3h**, **3i**, **3j**, **3k**), furnishing the target materials in yields ranging from 25% to 78%. Substrates featuring EDGs generally exhibited higher reaction efficiency, and a more pronounced electronic effect (whether donating or withdrawing) correlated with a greater influence on the yield. Notably, steric hindrance was found to have a notable influence on the reaction efficiency. Specifically, substrates with substituents at the *ortho*-position (**3b**, **3c**) proved to be recalcitrant, yielding a maximum of 39%. Similarly, the parallel trend was noted for the amide substrates bearing such substituents, reinforcing the pivotal role of electronic effects (**3k**–**3o**). Ulteriorly, investigation on directing group scopes demonstrated that while modifications such as substituted pyridines, quinoline, and isoquinoline were all viable, heterocycles like furan and thiophene were completely ineffective in promoting the transformation (**3p**–**3u**). Unfortunately, alkyl sulfides, benzyl sulfides, *N*-(2-ethylphenyl)picolinamide, and heteroaromatic amides like *N*-(3-methylpyridin-4-yl)picolinamide and *N*-(4-methylisoquinolin-3-yl)picolinamide are not applicable to this reaction, indicating a limited scope of the method (**3v**–**3z**). This transformation is highly sensitive to both steric and electronic parameters. The facilitatory effect of EDGs and the deleterious impact of ortho-substituents and certain heterocycles highlight this dependence. Most tellingly, the incompatibility of non-aromatic sulfides and specific amides defines a narrow substrate profile, suggesting a mechanism that demands precise electronic character and possibly chelation ability from the substrates. 

**Figure 2:**
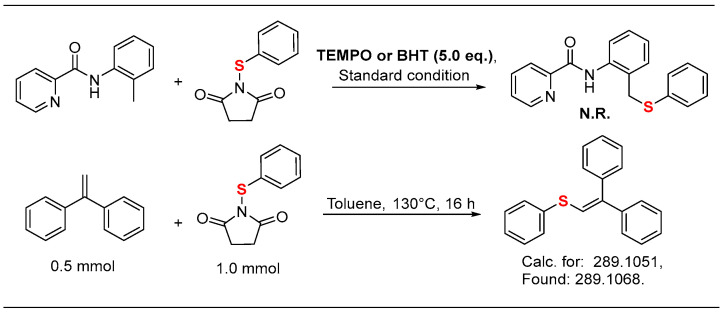
Mechanistic studies.

To gain more insights into the reaction mechanism, we incorporated 5.0 equivalents TEMPO or BHT into the reaction, and no desired products were obtained. Subsequently, the thioether radical was successfully trapped by 1,1-diphenylethylene and detected by ESI-HRMS (Figure 2).

**Figure 3:**
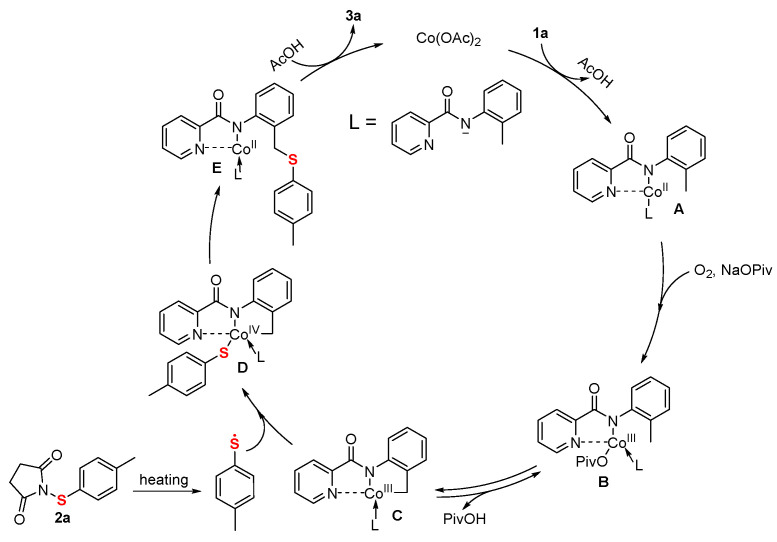
Proposed mechanism pathway.

Based on the experimental results presented and supported by previous literature [25,28,29,30,31], a plausible mechanism for this transformation via a Co^II^/Co^IV^ pathway is proposed (Figure 3). The catalytic cycle is initiated by the subsequent ligand exchange of Co(OAc)_2_ to the nitrogen atoms of *N*-(*o*-tolyl)picolinamide (**1a**) and forming the complex **A**. Then, **A** oxidized by O_2_ to yield cobalt(III) intermediate **B**, which undergoes reversible C-H cobaltation to provide cobaltacycle species **C**. Concurrently, toluylthiether radical was generated from 1-(*p*-tolylthio)pyrrolidine-2,5-dione (**2a**) upon heating and coupling with **C** to afford the intermediate **D.** Subsequent reorganization leads to complex **E**, and reductive elimination from E finally releases product (**3a**) and regenerates the cobalt catalyst for the next catalytic cycle.

## 3. Conclusions

In conclusion, we have developed a novel cobalt-catalyzed cross-coupling strategy for the picolinamide-directed C(sp^3^)-H chalcogenation of alkylamines using *N*-(phenylsulfanyl)succinimides. This method facilitates the direct construction of C(sp^3^)-S bonds under mild conditions and exhibits a broad substrate scope with excellent functional group tolerance. Notably, the catalytic system accomplishes oxidative C-H transformation without the need for expensive or environmentally harmful oxidants, thereby offering a sustainable and efficient alternative for C-S bond formation.

## 4. Experimental Section

All the chemicals were obtained commercially and used without any prior purification. ^1^H NMR and ^13^C NMR spectra were recorded on Bruker Avance II 400 or 500 spectrometers. All products were isolated by short chromatography on a silica gel (200–300 mesh) column using petroleum ether (60–90 °C) and ethyl acetate, unless otherwise noted. All compounds were characterized by ^1^H NMR and ^13^C NMR, which are consistent with those reported in the literature.

General procedure for the synthesis of amide substrates (**1**) [32,33]

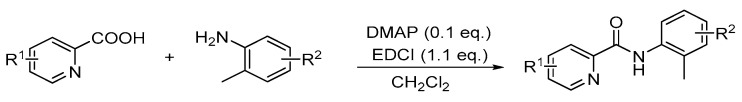

The typical procedure to make them: A 100 mL two-necked round-bottom flask was equipped with magnetic stir bar and charged with amine (20 mmol), picolinic acid (1.1 equiv), *N,N*-dimethyl-4-aminopyridine (DMAP, 0.1 equiv) dissolved in 30 mL anhydrous CH_2_Cl_2_ at 0 °C. Then EDCI (1.1 equiv) in CH_2_Cl_2_ (20 mL) was dropwise added to the solution under a nitrogen atmosphere. After the addition, the reaction was then warmed to room temperature, stirred for 12h and quenched with water (30 mL). Then the reaction mixture was extracted with ethyl acetate (20 × 3 mL) and the combined organic solvent was dried over Na_2_SO_4_, filtered and concentrated under reduced pressure. The resulting residue was purified by column chromatography using PE/AcOEt (15:1) as an eluent.

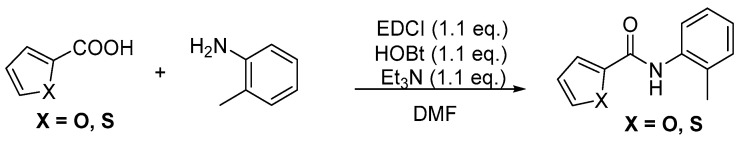



The typical procedure to make them: A 100 mL two-necked round-bottom flask was equipped with magnetic stir bar and charged with amine (5 mmol), acid (1.1 equiv), 1-ethyl-3-(3-dimethylaminopropyl)carbodiimide hydrochloride (EDCI, 5.5 mmol, 1.1 equiv.) and 1-Hydroxybenzotriazole (EDCI, 5.5 mmol, 1.1 equiv.) dissolved in 10 mL DMF. The reaction mixture was stirred at room temperature for 12 h, then poured into H_2_O (50 mL). The mixture was washed with 1.0 M HCl (15 mL), saturated aqueous NaHCO_3_ (15 mL), and brine (15 mL). Then the reaction mixture was extracted with ethyl acetate (20 × 3 mL) and the combined organic solvent was dried over Na_2_SO_4_, filtered and concentrated under reduced pressure. The resulting residue was purified by column chromatography using PE/AcOEt (15:1) as an eluent.

Preparation of *N*-aryl/alkylthiosuccinimide (**2**) [34]

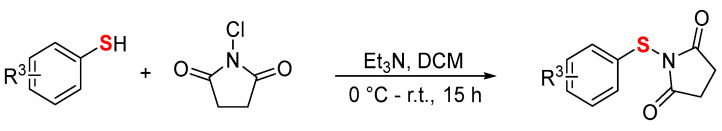

To a solution of *N*-chlorosuccinimide (NCS) (1.0 equiv) in CH_2_Cl_2_ (5.0 mL for 2.0 mmol) was added thiophenols (1.0 equiv) and Et_3_N (1.0 equiv) drop-wise under an argon atmosphere at 0 °C. The resulting mixture was stirred for 12 h at rt. After completion, the reaction mixture was quenched with saturated aqueous NH_4_Cl solution. The organic layer was separated; the aqueous layer was extracted with CH_2_Cl_2_ (20 × 3 mL). The combined extracts were washed with brine. The organic layer was dried over Na_2_SO_4_. Solvent was filtered and evaporated under reduced pressure. The crude residue was purified using column chromatography on silica gel using PE/AcOEt (5:1) as an eluent.

General procedure for synthesis of 3: A mixture of **1** (0.2 mmol), **2** (1.5 equiv.), Co(OAc)_2_·4H_2_O (20 mol%), NaOPiv (2.0 equiv), toluene (2.0 mL), stirred at 130 °C, under air, 24 h. Then the mixture was cooled to room temperature and concentrated under vacuum after filtration. The product **3** was purified by silica gel column flash chromatography using PE/AcOEt (30:1) as an eluent.

*N*-(2-((*p*-tolylthio)methyl)phenyl)picolinamide (**3a**): Colourless liquid, 72% yield; ^1^H NMR (400 MHz, CDCl_3_) δ 10.56 (s, 1H), 8.53 (d, *J* = 4.4 Hz, 1H), 8.23 (d, *J* = 7.8 Hz, 1H), 8.15 (d, *J* = 8.1 Hz, 1H), 7.83 (t, *J* = 7.2 Hz, 1H), 7.42–7.39 (m, 1H), 7.31 (d, *J* = 7.9 Hz, 2H), 7.26 (d, *J* = 7.6 Hz, 1H), 7.07 (d, *J* = 7.2 Hz, 1H), 7.01 (d, *J* = 8.2 Hz, 2H), 6.97 (d, *J* = 7.4 Hz, 1H), 4.08 (s, 2H), 2.24 (s, 3H). ^13^C NMR (101 MHz, CDCl_3_) δ 162.50, 150.22, 148.27, 137.73, 137.68, 136.24, 132.71, 131.41, 130.62, 129.81, 128.68, 127.82, 126.55, 124.78, 123.11, 122.63, 38.21, 21.26. HRMS(ESI+): Calculated for C_20_H_19_N_2_OS^+^, [M+H]^+^ 335.1218. Found 335.1221.

*N*-(2-(((2-chlorophenyl)thio)methyl)phenyl)picolinamide (**3c**): Obtained as a colourless liquid, 39% yield; ^1^H NMR (400 MHz, CDCl_3_) δ 10.49 (s, 1H), 8.44 (d, *J* = 4.0 Hz, 1H), 8.21 (d, *J* = 7.7 Hz, 1H), 8.11 (d, *J* = 8.0 Hz, 1H), 7.82 (d, *J* = 7.5 Hz, 1H), 7.39–7.36 (m, 1H), 7.35–7.29 (m, 2H), 7.26 (d, *J* = 7.6 Hz, 1H), 7.14 (d, *J* = 7.5 Hz, 1H), 7.10–7.06 (m, 2H), 6.99 (s, 1H), 4.16 (s, 2H). ^13^C NMR (101 MHz, CDCl_3_) δ 162.54, 150.04, 148.24, 137.66, 136.27, 135.89, 134.42, 132.34, 130.60, 129.83, 128.88, 128.23, 127.23, 127.08, 126.55, 124.97, 123.30, 122.55, 77.48, 77.16, 76.84, 35.55. HRMS(ESI+): Calculated for C_19_H_16_ClN_2_OS^+^, [M+H]^+^ 355.0672. Found 355.0679.

*N*-(2-((*m*-tolylthio)methyl)phenyl)picolinamide (**3d**): Colourless liquid, 62% yield; ^1^H NMR (400 MHz, CDCl_3_) δ 10.54 (s, 1H), 8.46 (d, *J* = 4.4 Hz, 1H), 8.21 (d, *J* = 7.7 Hz, 1H), 8.13 (d, *J* = 8.1 Hz, 1H), 7.79 (d, *J* = 7.7 Hz, 1H), 7.38–7.35 (m, 1H), 7.26 (t, *J* = 7.5 Hz, 1H), 7.19 (s, 1H), 7.12–7.06 (m, 2H), 7.01–6.91 (m, 3H), 4.10 (s, 2H), 2.20 (s, 3H). ^13^C NMR (101 MHz, CDCl_3_) δ 162.50, 150.17, 148.24, 138.77, 137.67, 136.26, 134.98, 132.53, 130.61, 128.89, 128.83, 128.72, 128.25, 127.67, 126.53, 124.81, 123.13, 122.60, 37.50, 21.39. HRMS(ESI+): Calculated for C_20_H_19_N_2_OS^+^, [M+H]^+^ 335.1218. Found 335.1210.

*N*-(2-(((3-bromophenyl)thio)methyl)phenyl)picolinamide (**3e**): Colourless liquid, 50% yield; ^1^H NMR (400 MHz, CDCl_3_) δ 10.51 (s, 1H), 8.52 (d, *J* = 4.5 Hz, 1H), 8.23 (d, *J* = 7.8 Hz, 1H), 8.15 (d, *J* = 8.1 Hz, 1H), 7.86–7.81 (m, 1H), 7.61 (s, 1H), 7.41 (dd, *J* = 7.0, 5.1 Hz, 1H), 7.31–7.28 (m, 3H), 7.14 (d, *J* = 7.2 Hz, 1H), 7.08 (d, *J* = 7.9 Hz, 1H), 7.05–7.01 (m, 1H), 4.15 (s, 2H). ^13^C NMR (101 MHz, CDCl_3_) δ 162.46, 150.03, 148.86, 148.37, 137.74, 136.03, 134.21, 130.61, 130.43, 130.33, 130.16, 129.06, 126.95, 126.65, 124.96, 123.27, 122.65, 120.39, 37.49. HRMS(ESI+): Calculated for C_19_H_16_BrN_2_OS^+^, [M+H]^+^ 399.0167. Found 399.0176.

*N*-(2-(((3-nitrophenyl)thio)methyl)phenyl)picolinamide (**3f**): Colourless liquid, 27% yield; ^1^H NMR (400 MHz, CDCl_3_) δ 10.42 (s, 1H), 8.47 (d, *J* = 4.4 Hz, 1H), 8.26–8.20 (m, 2H), 8.09 (d, *J* = 8.1 Hz, 1H), 8.00–7.94 (m, 1H), 7.85–7.81 (m, 1H), 7.61 (d, *J* = 7.7 Hz, 1H), 7.43–7.39 (m, 1H), 7.36 (t, *J* = 8.1 Hz, 1H), 7.30 (t, *J* = 7.7 Hz, 1H), 7.16 (s, 1H), 7.04 (d, *J* = 7.5 Hz, 1H), 4.22 (s, 2H). ^13^C NMR (101 MHz, CDCl_3_) δ 162.46, 148.32, 137.79, 137.00, 132.47, 130.61, 129.70, 129.22, 126.74, 125.66, 125.18, 123.59, 122.65, 122.02, 121.73, 117.97, 114.82, 113.91, 36.99. HRMS(ESI+): Calculated for C_19_H_16_N_3_O_3_S^+^, [M+H]^+^ 366.0912. Found 366.0920.

*N*-(2-(((4-methoxyphenyl)thio)methyl)phenyl)picolinamide (**3g**): Colourless liquid, 78% yield; ^1^H NMR (400 MHz, CDCl_3_) δ 10.54 (s, 1H), 8.61–8.54 (m, 1H), 8.24 (d, *J* = 7.8 Hz, 1H), 8.16 (d, *J* = 8.0 Hz, 1H), 7.84 (td, *J* = 7.7, 1.7 Hz, 1H), 7.44–7.39 (m, 1H), 7.37–7.31 (m, 2H), 7.29–7.23 (m, 1H), 7.01–6.93 (m, 2H), 6.76–6.68 (m, 2H), 4.02 (s, 2H), 3.70 (s, 3H). ^13^C NMR (101 MHz, CDCl_3_) δ 162.43, 159.82, 150.18, 148.22, 137.70, 136.12, 135.45, 130.65, 128.57, 128.00, 126.56, 125.07, 124.70, 123.06, 122.64, 114.58, 55.42, 39.14. HRMS(ESI+): Calculated for C_20_H_19_N_2_O_2_S^+^, [M+H]^+^ 351.1167. Found 351.1158.

*N*-(2-(((4-chlorophenyl)thio)methyl)phenyl)picolinamide (**3h**): Colourless liquid, 52% yield; ^1^H NMR (400 MHz, CDCl_3_) δ 10.38 (s, 1H), 8.40 (d, *J* = 4.4 Hz, 1H), 8.14 (d, *J* = 7.8 Hz, 1H), 8.04 (d, *J* = 8.1 Hz, 1H), 7.77–7.73 (m, 1H), 7.32 (dd, *J* = 6.8, 5.0 Hz, 1H), 7.21 (t, *J* = 6.0 Hz, 3H), 7.09 (d, *J* = 5.9 Hz, 2H), 6.99 (d, *J* = 7.0 Hz, 1H), 6.91 (t, *J* = 7.3 Hz, 1H), 4.01 (s, 2H). ^13^C NMR (101 MHz, CDCl_3_) δ 162.44, 150.06, 148.20, 137.75, 136.16, 133.63, 133.40, 130.58, 129.15, 128.90, 127.43, 126.63, 124.94, 123.31, 122.66, 37.70. HRMS(ESI+): Calculated for C_19_H_16_ClN_2_OS^+^, [M+H]^+^ 355.0672. Found 355.0677.

*N*-(2-(((4-bromophenyl)thio)methyl)phenyl)picolinamide (**3i**): Colourless liquid, 59% yield; ^1^H NMR (400 MHz, CDCl_3_) δ 10.46 (s, 1H), 8.47 (d, *J* = 4.4 Hz, 1H), 8.22 (d, *J* = 7.8 Hz, 1H), 8.12 (d, *J* = 8.1 Hz, 1H), 7.86–7.81 (m, 1H), 7.40 (dd, *J* = 6.8, 5.1 Hz, 1H), 7.30 (t, *J* = 7.8 Hz, 3H), 7.23 (d, *J* = 8.4 Hz, 2H), 7.08 (d, *J* = 7.0 Hz, 1H), 7.00 (t, *J* = 7.3 Hz, 1H), 4.10 (s, 2H). ^13^C NMR (101 MHz, CDCl_3_) δ 162.44, 150.05, 148.23, 137.76, 136.17, 134.39, 133.50, 132.10, 130.58, 128.93, 127.40, 126.65, 124.97, 123.34, 122.67, 121.62, 77.48, 77.16, 76.84, 37.52. HRMS(ESI+): Calculated for C_19_H_16_BrN_2_OS^+^, [M+H]^+^ 399.0167. Found 399.0161.

*N*-(2-(((4-nitrophenyl)thio)methyl)phenyl)picolinamide (**3j**): Light yellow liquid, 25% yield; 1H NMR (400 MHz, CDCl3) δ 10.36 (s, 1H), 8.34 (d, *J* = 4.1 Hz, 1H), 8.21 (d, *J* = 7.7 Hz, 1H), 8.05 (d, *J* = 8.6 Hz, 3H), 7.83 (t, *J* = 7.5 Hz, 1H), 7.40 (d, *J* = 8.4 Hz, 3H), 7.34–7.26 (m, 2H), 7.08 (t, *J* = 7.4 Hz, 1H), 4.27 (s, 2H). 13C NMR (101 MHz, CDCl3) δ 162.43, 149.76, 148.17, 141.96, 137.89, 136.19, 130.48, 129.38, 128.42, 126.80, 126.61, 125.50, 124.06, 123.84, 122.71, 117.37, 77.48, 77.16, 76.84, 35.22. HRMS(ESI+): Calculated for C_19_H_16_N_3_O_3_S^+^, [M+H]^+^ 366.0912. Found 366.0922.

*N*-(5-methyl-2-((phenylthio)methyl)phenyl)picolinamide (**3k**): Colourless liquid, 57% yield; ^1^H NMR (400 MHz, CDCl_3_) δ 10.55 (s, 1H), 8.49 (d, *J* = 4.3 Hz, 1H), 8.25 (d, *J* = 7.6 Hz, 1H), 8.01 (s, 1H), 7.86 (d, *J* = 7.0 Hz, 1H), 7.45–7.40 (m, 3H), 7.24–7.19 (m, 2H), 7.16 (s, 1H), 7.03 (d, *J* = 7.7 Hz, 1H), 6.83 (d, *J* = 7.5 Hz, 1H), 4.14 (s, 2H), 2.32 (s, 3H). ^13^C NMR (101 MHz, CDCl_3_) δ 162.46, 150.20, 148.24, 138.82, 137.69, 136.02, 135.57, 131.73, 130.40, 129.00, 127.28, 126.52, 125.67, 124.49, 123.71, 122.58, 37.24, 21.56 HRMS(ESI+): Calculated for C_20_H_19_N_2_OS^+^, [M+H]^+^ 335.1218. Found 335.1228.

*N*-(2-(((4-chlorophenyl)thio)methyl)-5-methylphenyl)picolinamide (**3l**): Colourless liquid, 44% yield; ^1^H NMR (400 MHz, CDCl_3_) δ 10.48 (s, 1H), 8.50 (d, *J* = 4.3 Hz, 1H), 8.25 (d, *J* = 7.8 Hz, 1H), 8.00 (s, 1H), 7.87 (dd, *J* = 11.0, 4.3 Hz, 1H), 7.45–7.42 (m, 1H), 7.34 (d, *J* = 8.4 Hz, 2H), 7.22–7.19 (m, 2H), 7.00 (d, *J* = 7.7 Hz, 1H), 6.84 (d, *J* = 7.6 Hz, 1H), 4.10 (s, 2H), 2.33 (s, 3H). ^13^C NMR (101 MHz, CDCl_3_) δ 162.39, 150.06, 148.20, 138.98, 137.75, 135.90, 133.86, 133.48, 133.23, 130.40, 129.13, 126.61, 125.76, 124.29, 123.84, 122.61, 77.48, 77.16, 76.84, 37.44, 21.55. HRMS(ESI+): Calculated for C_20_H_18_ClN_2_OS^+^, [M+H]^+^ 369.0828. Found 369.0825.

*N*-(4-chloro-2-((phenylthio)methyl)phenyl)picolinamide (**3m**): Colourless liquid, 47% yield; ^1^H NMR (400 MHz, CDCl_3_) δ 10.52 (s, 1H), 8.48 (d, *J* = 4.5 Hz, 1H), 8.21 (d, *J* = 7.8 Hz, 1H), 8.12 (d, *J* = 8.7 Hz, 1H), 7.83 (t, *J* = 7.7 Hz, 1H), 7.42–7.38 (m, 3H), 7.24–7.20 (m, 3H), 7.18 (s, 1H), 7.06 (d, *J* = 2.3 Hz, 1H), 4.06 (s, 2H). ^13^C NMR (101 MHz, CDCl_3_) δ 162.48, 149.82, 148.29, 137.78, 134.80, 134.58, 132.21, 130.31, 129.71, 129.35, 129.15, 128.63, 127.80, 126.73, 124.20, 122.65, 37.24. HRMS(ESI+): Calculated for C_19_H_16_ClN_2_OS^+^, [M+H]^+^ 355.0672. Found 355.0679.

*N*-(3-chloro-2-(((4-methoxyphenyl)thio)methyl)phenyl)picolinamide (**3n**): Colourless liquid, 44% yield; ^1^H NMR (400 MHz, CDCl_3_) δ 10.46 (s, 1H), 8.55 (d, *J* = 4.4 Hz, 1H), 8.20 (d, *J* = 7.8 Hz, 1H), 8.05 (d, *J* = 8.1 Hz, 1H), 7.85–7.81 (m, 1H), 7.44–7.42 (m, 1H), 7.39 (d, *J* = 8.8 Hz, 2H), 7.16 (s, 1H), 7.09 (d, *J* = 7.9 Hz, 1H), 6.70 (d, *J* = 8.7 Hz, 2H), 4.21 (s, 2H), 3.69 (s, 3H). ^13^C NMR (101 MHz, CDCl_3_) δ 162.52, 160.12, 149.86, 148.23, 137.75, 137.69, 136.06, 134.96, 128.79, 126.74, 125.93, 124.52, 122.70, 121.70, 114.93, 114.61, 55.43, 35.28. HRMS(ESI+): Calculated for C_20_H_18_ClN_2_OS^+^, [M+H]^+^ 385.0778. Found 385.0782.

6-methyl-*N*-(2-((phenylthio)methyl)phenyl)picolinamide (**3p**): White solid, 59% yield; ^1^H NMR (400 MHz, CDCl_3_) δ 10.68 (s, 1H), 8.20 (d, *J* = 8.1 Hz, 1H), 8.07 (d, *J* = 7.6 Hz, 1H), 7.74 (t, *J* = 7.7 Hz, 1H), 7.36 (d, *J* = 7.2 Hz, 2H), 7.32–7.24 (m, 3H), 7.12–7.19 (m, 2H), 7.12 (d, *J* = 7.3 Hz, 1H), 7.02 (t, *J* = 7.4 Hz, 1H), 4.18 (s, 2H), 2.43 (s, 3H). ^13^C NMR (101 MHz, CDCl_3_) δ 162.68, 157.46, 149.37, 137.88, 136.37, 135.43, 130.98, 130.67, 129.01, 128.77, 127.32, 127.08, 126.30, 124.69, 123.07, 119.68, 36.74, 24.18. HRMS(ESI+): Calculated for C_20_H_19_N_2_OS^+^, [M+H]^+^ 335.1218. Found 335.1223.

*N*-(2-(((4-methoxyphenyl)thio)methyl)phenyl)-3-methylpicolinamide (**3q**): Colourless liquid, 61% yield; ^1^H NMR (400 MHz, CDCl_3_) δ 10.64 (s, 1H), 8.39 (d, *J* = 4.1 Hz, 1H), 8.08 (d, *J* = 8.1 Hz, 1H), 7.57 (d, *J* = 7.6 Hz, 1H), 7.31–7.28 (m, 3H), 7.24 (t, *J* = 7.3 Hz, 1H), 6.99–6.92 (m, 2H), 6.70 (d, *J* = 8.5 Hz, 2H), 4.00 (s, 2H), 3.70 (s, 3H), 2.74 (s, 3H). ^13^C NMR (101 MHz, CDCl_3_) δ 164.08, 159.79, 147.25, 145.65, 141.30, 136.42, 136.34, 135.33, 130.64, 128.46, 128.25, 126.12, 125.29, 124.54, 123.20, 114.59, 55.45, 39.09, 20.92. HRMS(ESI+): Calculated for C_21_H_21_N_2_O_2_S^+^, [M+H]^+^ 365.1324. Found 365.1328.

*N*-(2-(((4-methoxyphenyl)thio)methyl)phenyl)quinoline-2-carboxamide (**3r**): White solid, 69% yield; ^1^H NMR (400 MHz, CDCl_3_) δ 10.82 (s, 1H), 8.32 (q, *J* = 8.5 Hz, 2H), 8.19 (d, *J* = 8.0 Hz, 1H), 8.01 (d, *J* = 8.5 Hz, 1H), 7.84 (d, *J* = 7.5 Hz, 1H), 7.69–7.67 (m, 1H), 7.60–7.53 (m, 1H), 7.34–7.26 (m, 3H), 6.97 (d, *J* = 4.1 Hz, 2H), 6.71–6.66 (m, 2H), 4.08 (s, 2H), 3.68 (s, 3H). ^13^C NMR (101 MHz, CDCl_3_) δ 162.65, 159.74, 149.96, 146.52, 137.88, 136.23, 135.09, 130.79, 130.31, 130.14, 129.58, 128.63, 128.29, 128.03, 127.85, 125.07, 124.68, 123.03, 118.98, 114.64, 55.43, 38.84. HRMS(ESI+): Calculated for C_24_H_21_N_2_O_2_S^+^, [M+H]^+^ 401.1324. Found 401.1330

*N*-(2-(((4-methoxyphenyl)thio)methyl)phenyl)isoquinoline-3-carboxamide (**3s**): White solid, 72% yield; ^1^H NMR (400 MHz, CDCl_3_) δ 10.76 (s, 1H), 9.70–9.60 (m, 1H), 8.47 (d, *J* = 5.5 Hz, 1H), 8.16 (d, *J* = 8.1 Hz, 1H), 7.82–7.78 (m, 2H), 7.69–7.61 (m, 2H), 7.35–7.26 (m, 3H), 7.04–6.94 (m, 2H), 6.75–6.68 (m, 2H), 4.05 (s, 2H), 3.67 (s, 3H). ^13^C NMR (101 MHz, CDCl_3_) δ 164.13, 159.78, 147.92, 140.29, 137.74, 136.32, 135.34, 130.71, 128.99, 128.53, 128.50, 127.93, 127.48, 127.06, 125.14, 124.94, 124.77, 123.37, 114.58, 77.48, 77.16, 76.84, 55.41, 39.12. HRMS(ESI+): Calculated for C_24_H_21_N_2_O_2_S^+^, [M+H]^+^ 401.1324. Found 401.1329.

## Figures and Tables

**Table 1:** Optimization of reaction conditions ^a,b^.

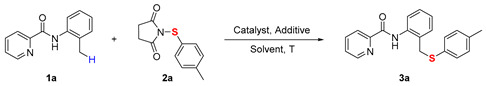				
Entry	Catalyst	Additive	Solvent	Yield [%] ^b^
1	Co(OAc)_2_·4H_2_O	KOAc	Toluene	57
2	CoBr_2_	KOAc	Toluene	48
3	Co(acac)_2_	KOAc	Toluene	trace
4	NiCl_2_	KOAc	Toluene	N.R
5	CuBr_2_	KOAc	Toluene	N.R.
6	Cu(OAc)_2_	KOAc	Toluene	N.R.
7	Fe(OAc)_2_	KOAc	Toluene	trace
8	-	KOAc	Toluene	N.R.
9	Co(OAc)_2_·4H_2_O	NaOAc	Toluene	47
10	Co(OAc)_2_·4H_2_O	Na_2_CO_3_	Toluene	39
11	Co(OAc)_2_·4H_2_O	K_2_CO_3_	Toluene	33
12	Co(OAc)_2_·4H_2_O	NaOPiv	Toluene	72
13	Co(OAc)_2_·4H_2_O	-	Toluene	42
14	Co(OAc)_2_·4H_2_O	NaOPiv	*p*-xylene	58
15	Co(OAc)_2_·4H_2_O	NaOPiv	DMSO	43
16	Co(OAc)_2_·4H_2_O	NaOPiv	DMF	48
17	Co(OAc)_2_·4H_2_O	NaOPiv	Mesitylene	61
18	Co(OAc)_2_·4H_2_O	NaOPiv	Toluene	60 ^c^
19	Co(OAc)_2_·4H_2_O	NaOPiv	Toluene	13 ^d^
20	Co(OAc)_2_·4H_2_O	NaOPiv	Toluene	76 ^e^

[a] Reaction conditions: **1a** (0.2 mmol), **2a** (1.5 equiv.), catalyst (20 mol%), additive (2.0 equiv), solvent (2.0 mL), stirred at 130 °C, under air, 24 h. [b] Isolated yields. [c] Co(OAc)_2_ (10 mol%). [d] Under N_2_ atmosphere. [e] Under O_2_ atmosphere.

**Table 2:** Scope of sulfurization substrates ^a,b^.

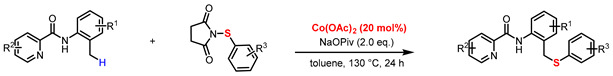
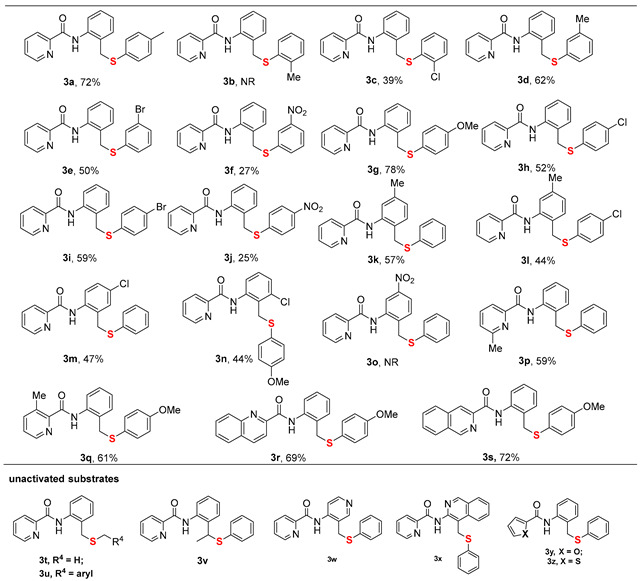

[a] Reaction conditions: **1** (0.2 mmol), **2** (1.5 equiv.), Co(OAc)_2_·4H_2_O (20 mol%), NaOPiv (2.0 equiv), toluene (2.0 mL), stirred at 130 °C, under air, 24 h. [b] Isolated yields.

## Data Availability

The original contributions presented in this study are included in the article/Supplementary Materials. Further inquiries can be directed to the corresponding authors.

## Supplementary Materials

The following supporting information can be downloaded at: https://www.mdpi.com/article/10.3390/molecules30224462/s1, Figure S1: ^1^H NMR Spectra of Compound **3a**; Figure S2: ^13^C NMR Spectra of Compound **3a**; Figure S3: ^1^H NMR Spectra of Compound **3c**; Figure S4:^13^C NMR Spectra of Compound **3c**; Figure S5: ^1^H NMR Spectra of Compound **3d**; Figure S6: ^13^CNMR Spectra of Compound **3d**; Figure S7: ^1^H NMR Spectra of Compound **3e**; Figure S8: ^13^C NMR Spectra of Compound **3e**; Figure S9: ^1^H NMR Spectra of Compound **3f**; Figure S10: ^13^C NMR Spectra of Compound **3f**; Figure S11: ^1^H NMR Spectra of Compound **3g**; Figure S12: ^13^C NMR Spectra of Compound **3g**; Figure S13: ^1^H NMR Spectra of Compound **3h**; Figure S14: ^13^C NMR Spectra of Compound **3h**; Figure S15: ^1^H NMR Spectra of Compound **3i**; Figure S16: ^13^C NMR Spectra of Compound **3i**; Figure S17: ^1^H NMR Spectra of Compound **3j**; Figure S18: ^13^C NMR Spectra of Compound **3j**; Figure S19: ^1^H NMR Spectra of Compound **3k**; Figure S20: ^13^C NMR Spectra of Compound **3k**; Figure S21: ^1^H NMR Spectra of Compound **3l**; Figure S22: ^13^C NMR Spectra of Compound **3l**; Figure S23: ^1^H NMR Spectra of Compound **3m**; Figure S24: ^13^C NMR Spectra of Compound **3m**; Figure S25: ^1^H NMR Spectra of Compound **3n**; Figure S26: ^13^C NMR Spectra of Compound **3n**; Figure S27: ^1^H NMR Spectra of Compound **3p**; Figure S28: ^13^C NMR Spectra of Compound **3p**; Figure S29: ^1^H NMR Spectra of Compound **3q**; Figure S30: ^13^C NMR Spectra of Compound **3q**; Figure S31: ^1^H NMR Spectra of Compound **3r**; Figure S32: ^13^C NMR Spectra of Compound **3r**; Figure S33: ^1^H NMR Spectra of Compound **3s**; Figure S34: ^13^C NMR Spectra of Compound **3s**.
