# Supplementary material for: Co(II)-Catalyzed Picolinamide-Directed C(sp3)-S Bond Formation with N-(phenylsulfanyl)succinimides
Source: Molecules. 2025 Nov 19;30(22):4462. doi: 10.3390/molecules30224462 (PMC12655464; doi:10.3390/molecules30224462)

Supporting Information

Co(II)-Catalyzed Picolinamide-Directed C(sp^3)-S Bond Formation with *N*-(phenylsulfanyl)succinimides

Jinjing Qin [1,2], Shaodong Zhou [1], Jinwen Luo [2], Guodong Wang [3,*] and Kai Wang [4,*]

[1] College of Chemical and Biological Engineering, Zhejiang University, Hangzhou 310058, China
[2] Zhejiang Anglikang Pharmaceutical Co., Ltd., Shengzhou 312400, China
[3] School of Pharmacy, Qilu Medical University, Zibo 255300, China
[4] College of Medical Engineering, Jining Medical University, Jining 272002, China
* Correspondence: wanggd1991@163.com (G.W.); wangkaichem@mail.jnmc.edu.cn (K.W.)

Table of Contents

1. Copies of ^{1}H and ^{13}C NMR Spectra 2

1. Copies of ^1H and ^{13}C NMR Spectra

Figure S1 ^1H NMR Spectra of Compound of 3a

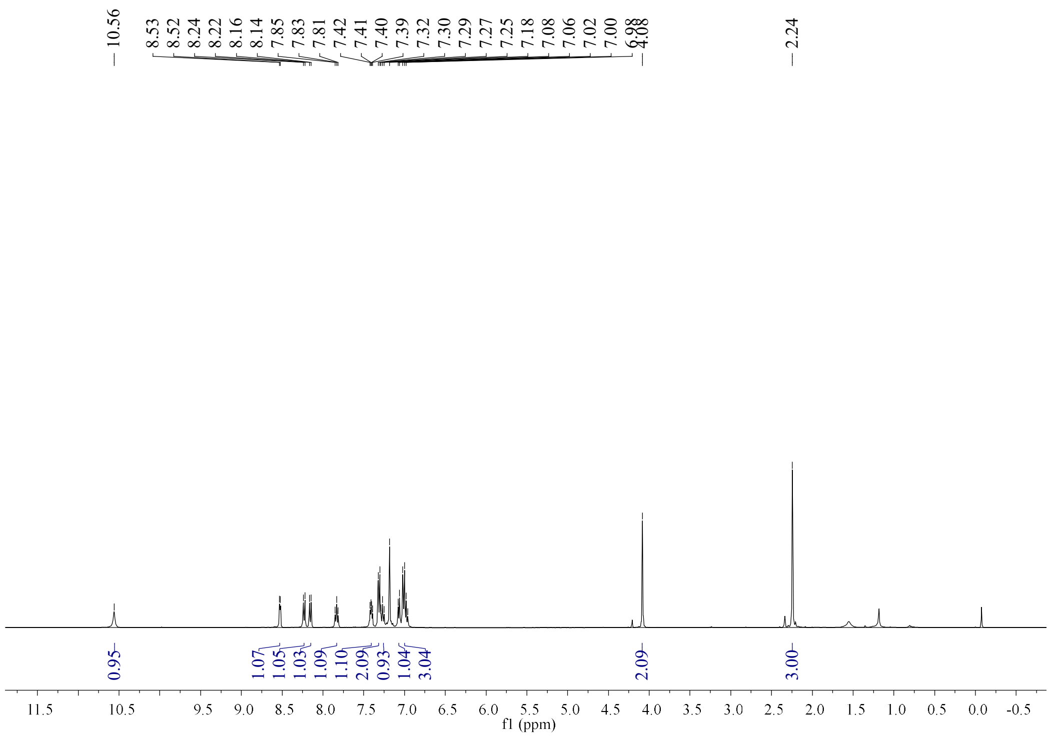

Figure S2 ^{13}C NMR Spectra of Compound of 3a

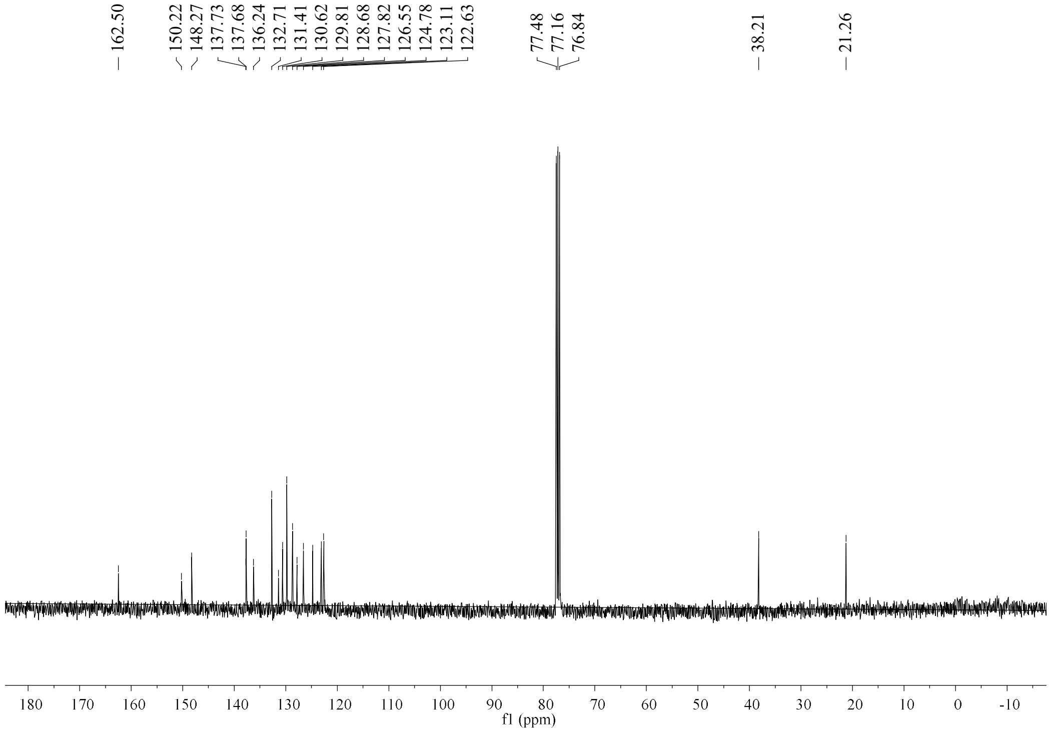

Figure S3 ^{1}H NMR Spectra of Compound of 3c

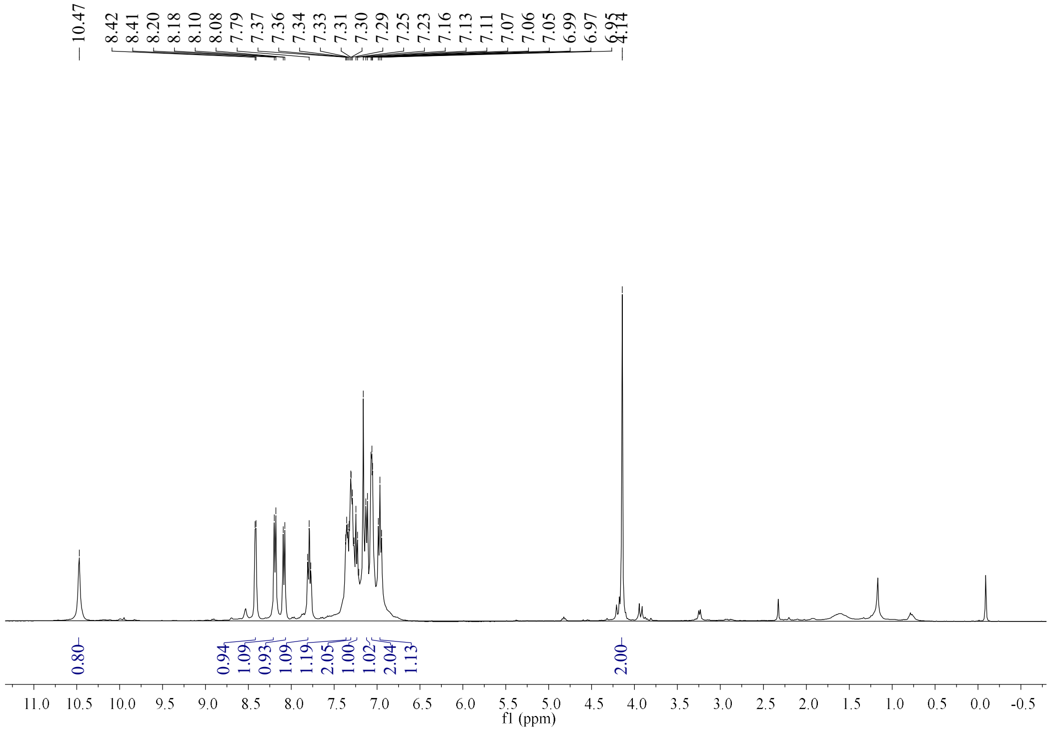

Figure S4 ^{13}C NMR Spectra of Compound of 3c

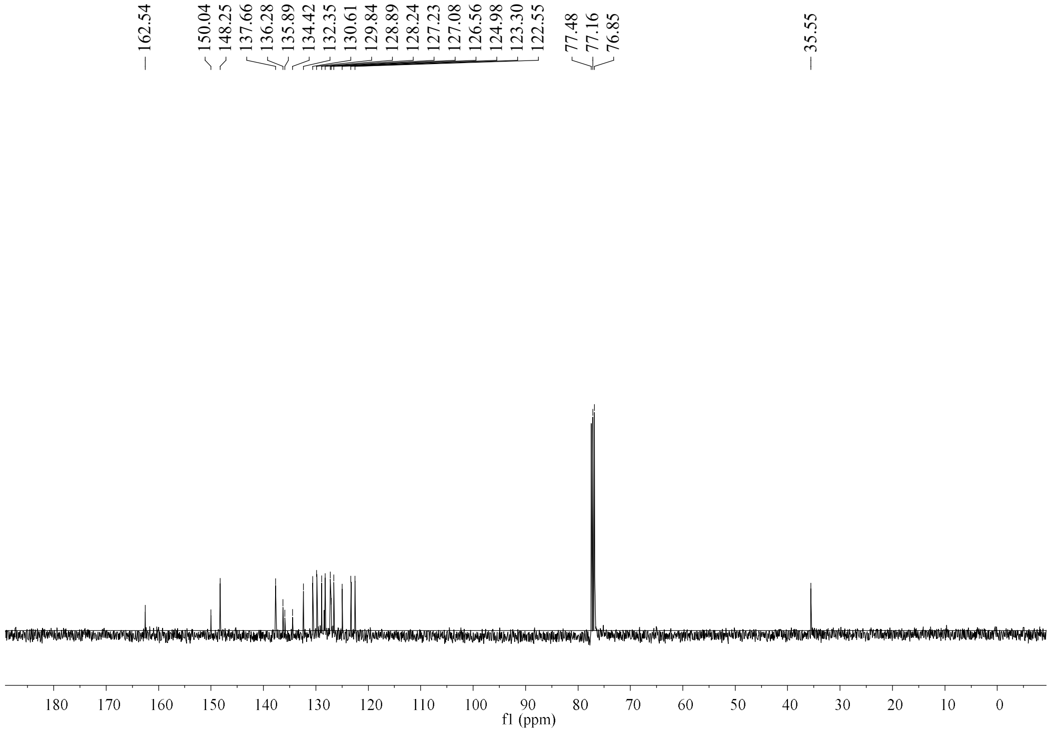

Figure S5 ^{1}H NMR Spectra of Compound of 3d

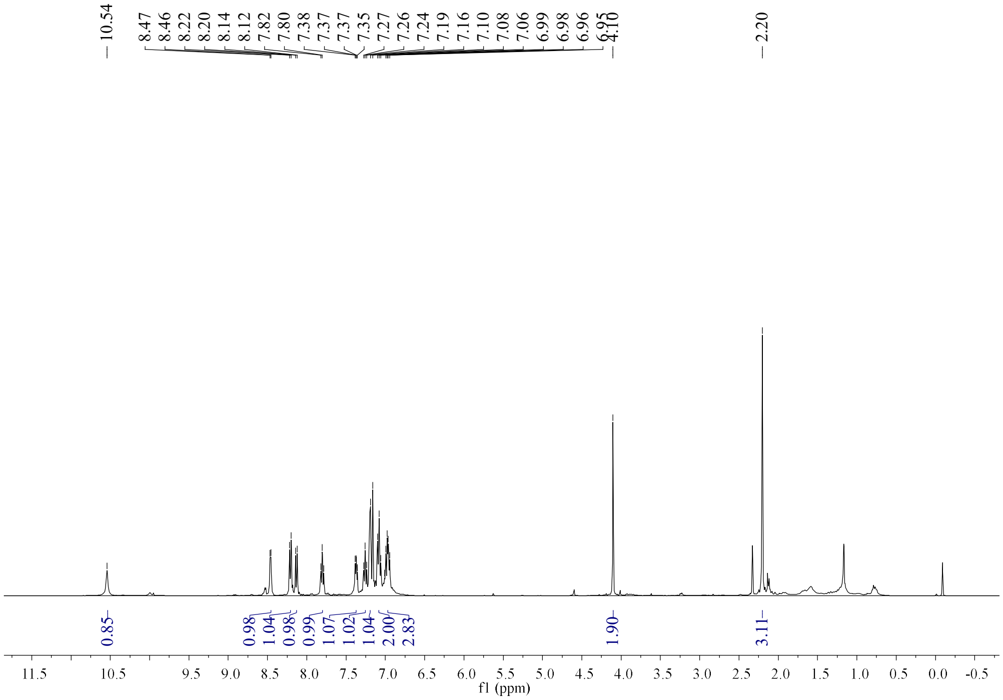

Figure S6 ^{13}C NMR Spectra of Compound of 3d

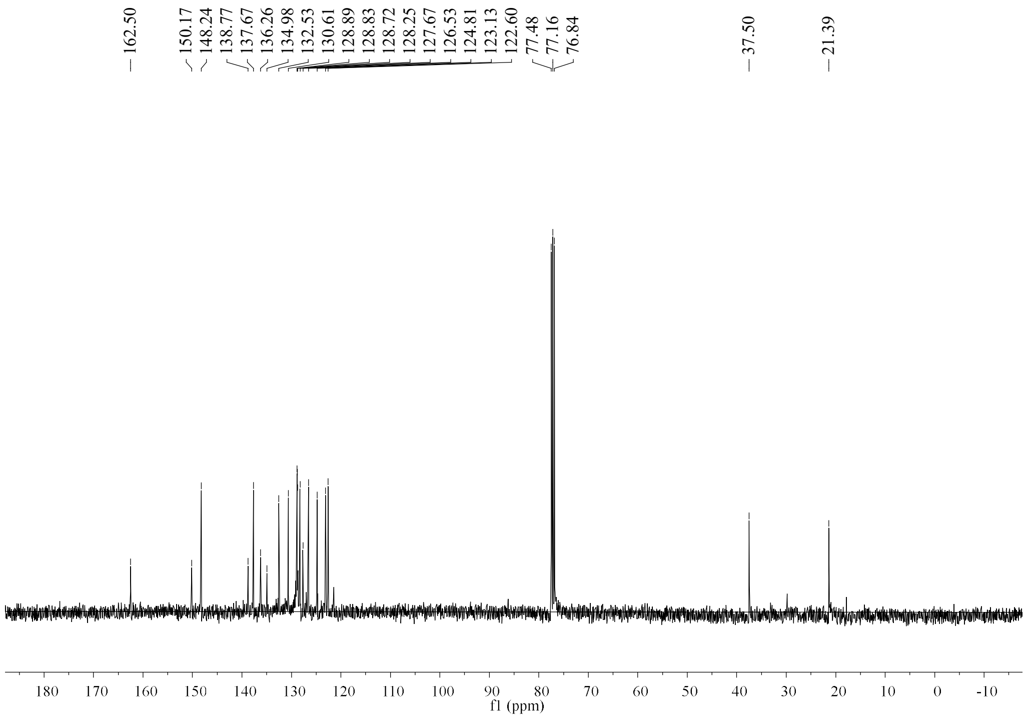

Figure S7 ^{1}H NMR Spectra of Compound of 3e

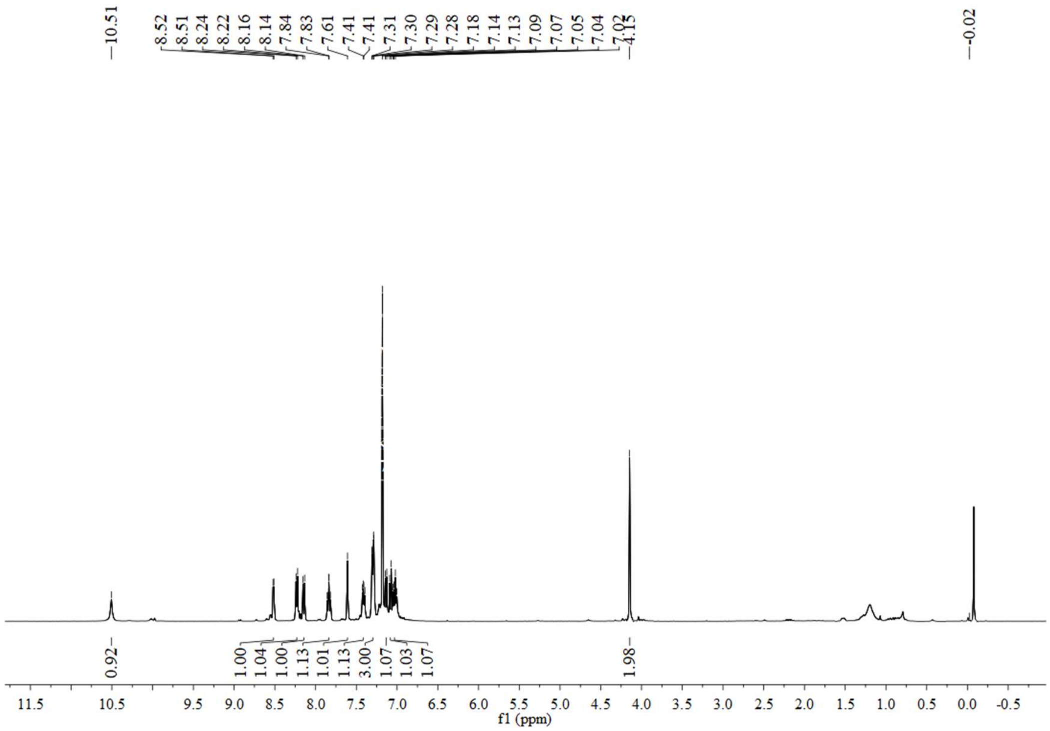

Figure S8 ^{13}C NMR Spectra of Compound of 3e

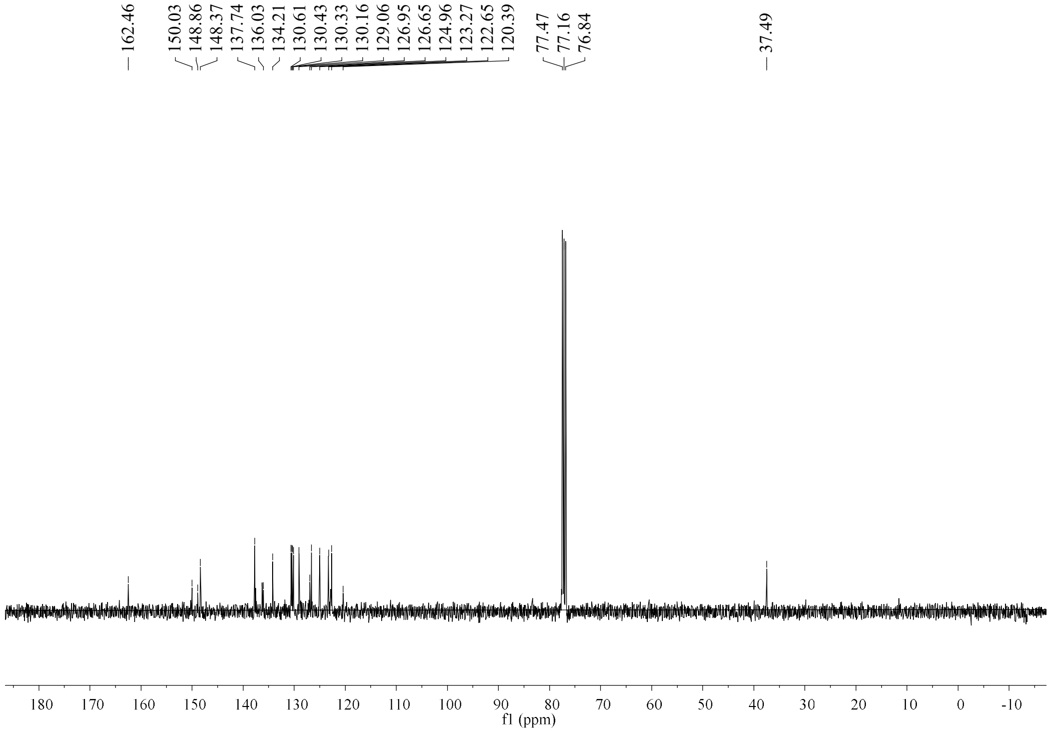

Figure S9 ^{1}H NMR Spectra of Compound of 3f

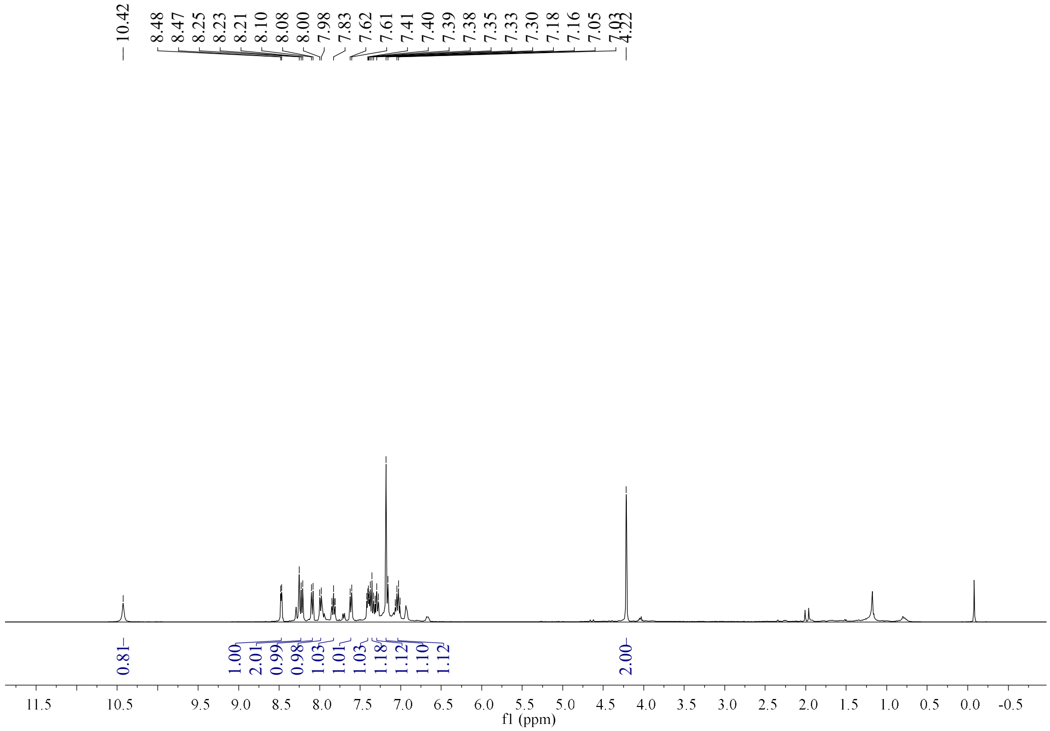

Figure S10 ^{13}C NMR Spectra of Compound of 3f

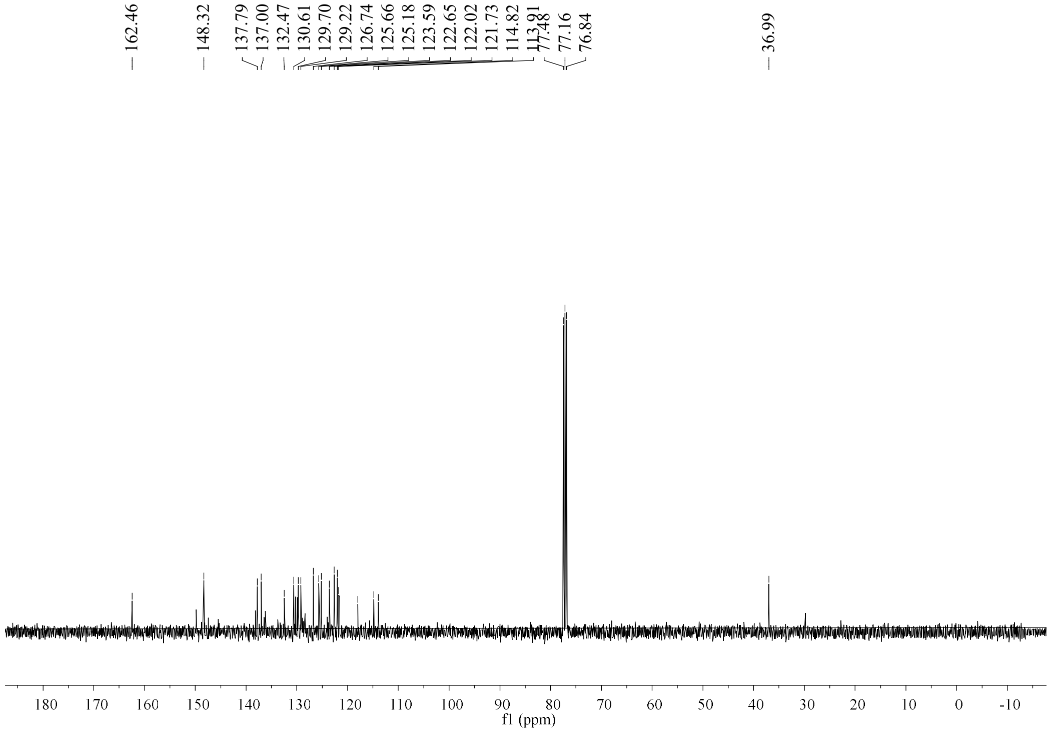

Figure S11 ^{1}H NMR Spectra of Compound of 3g

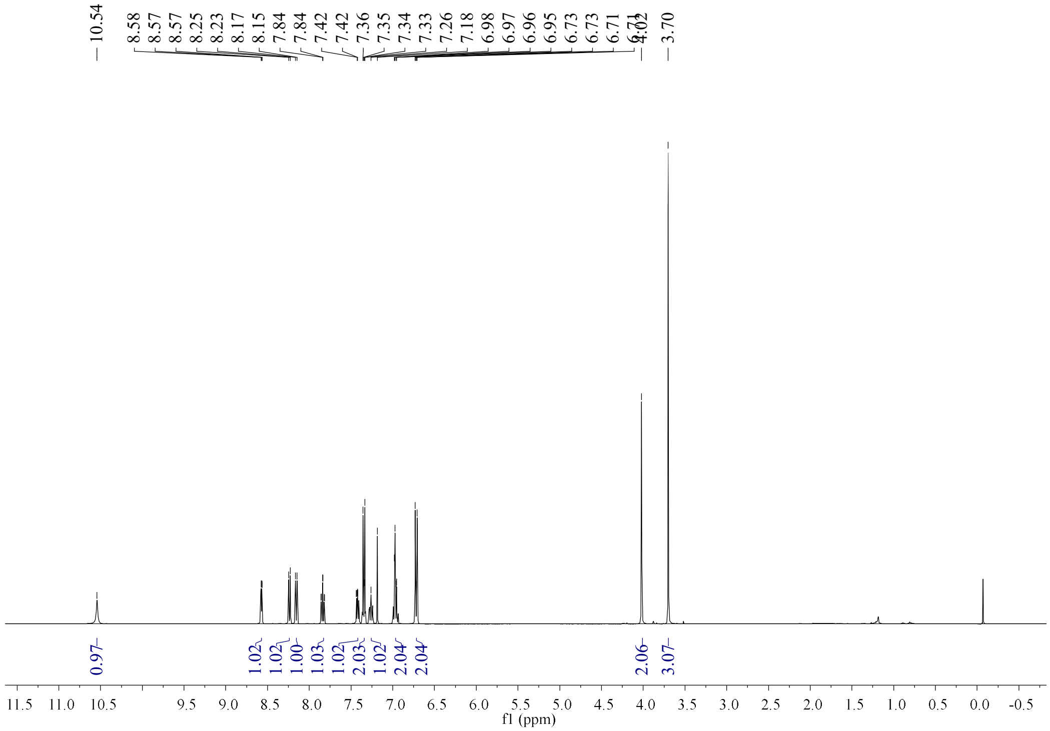

Figure S12 ^{13}C NMR Spectra of Compound of 3g

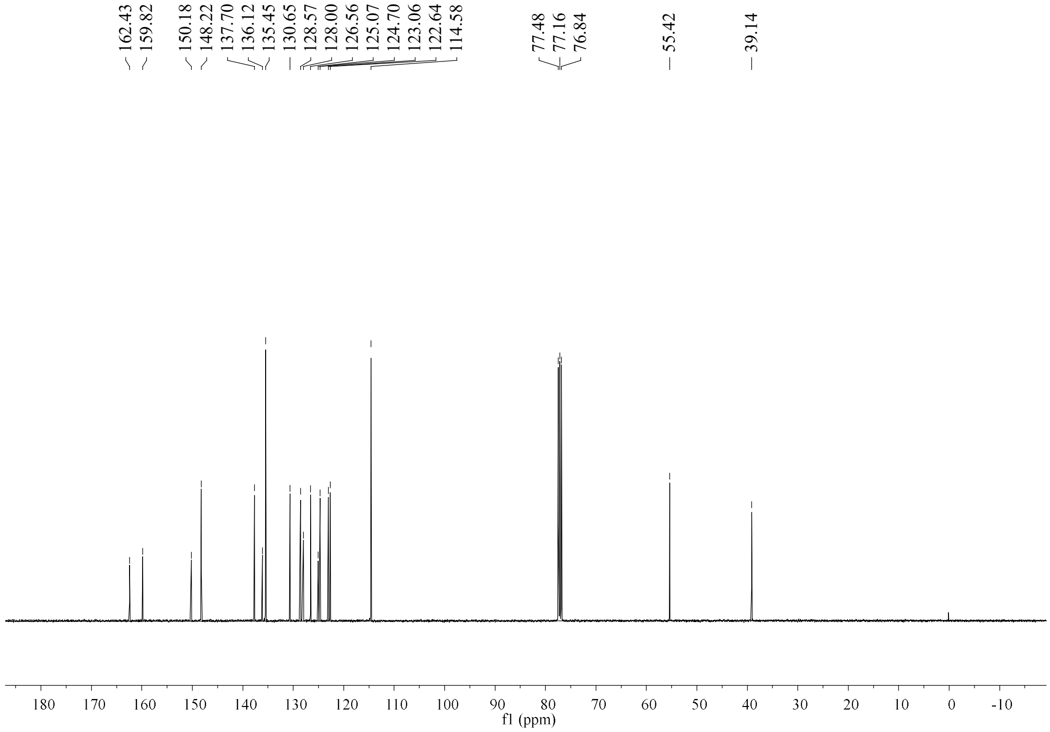

Figure S13 ^{1}H NMR Spectra of Compound of 3h

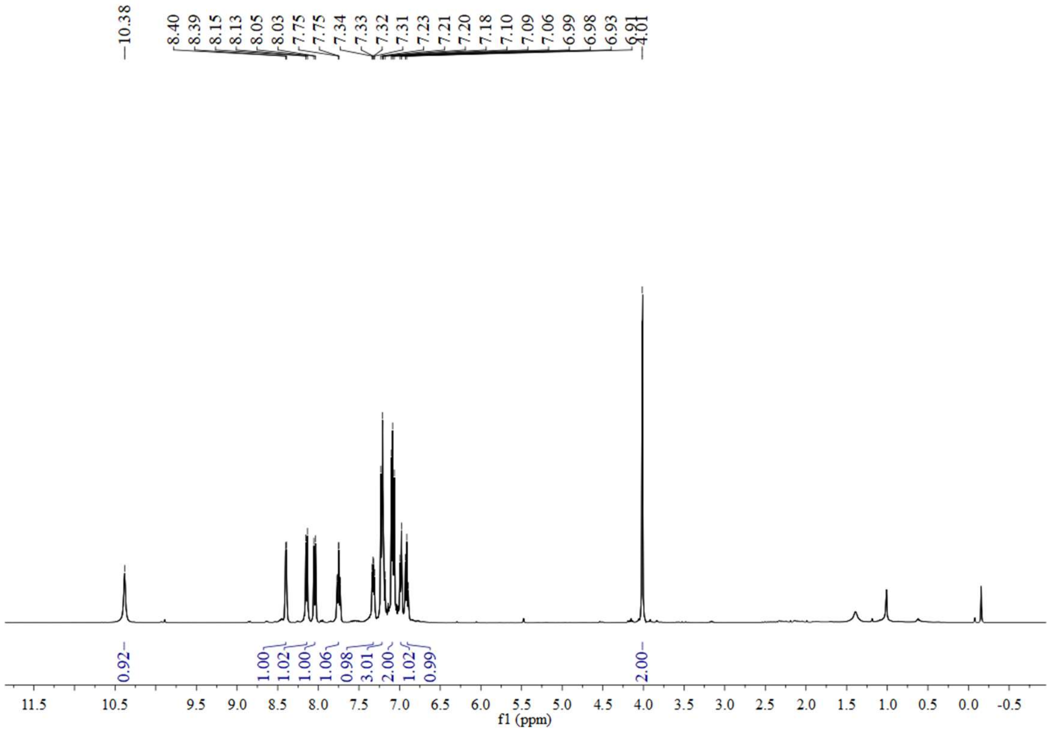

Figure S14 ^{13}C NMR Spectra of Compound of 3h

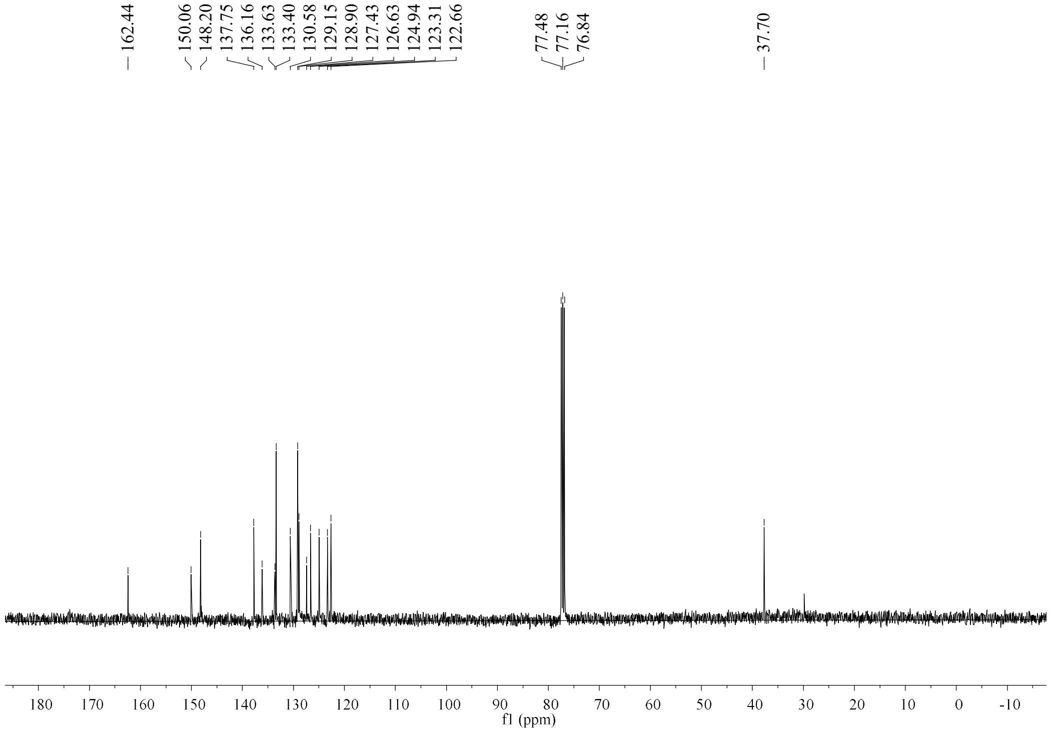

Figure S15 ^{1}H NMR Spectra of Compound of 3i

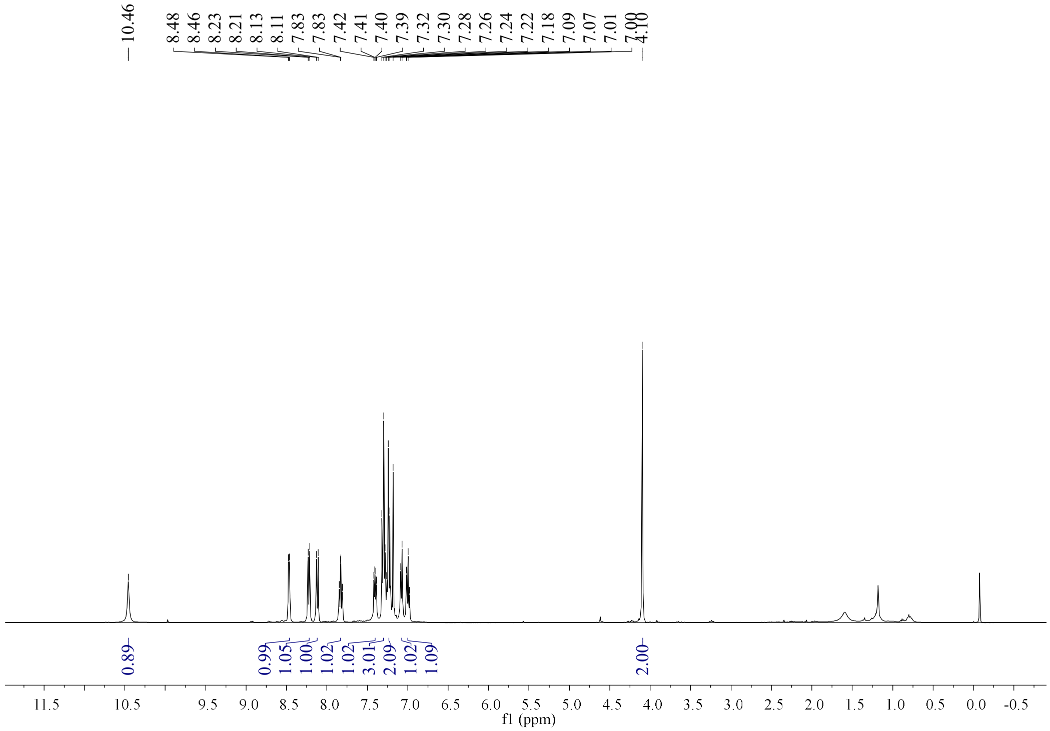

Figure S16 ^{13}C NMR Spectra of Compound of 3i

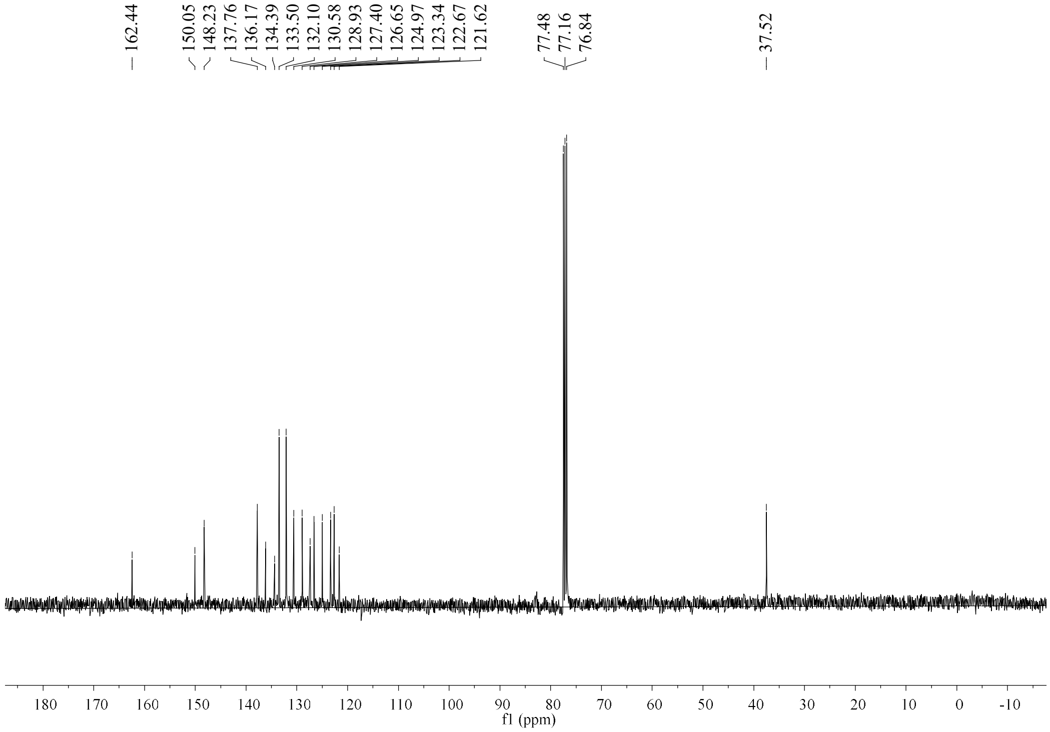

Figure S17 ^{1}H NMR Spectra of Compound of 3j

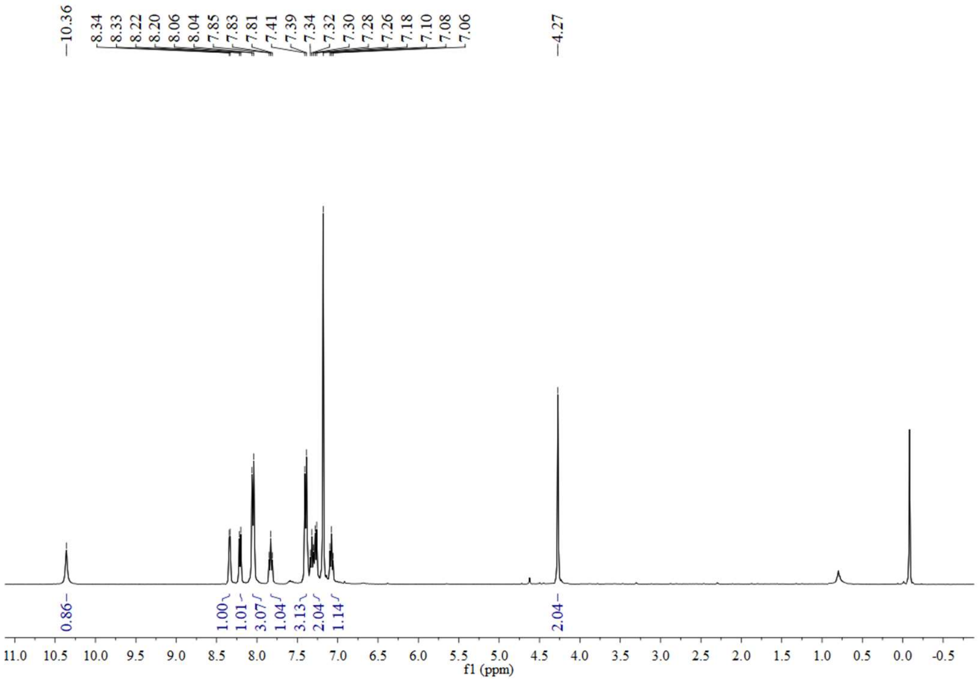

Figure S18 ^{13}C NMR Spectra of Compound of 3j

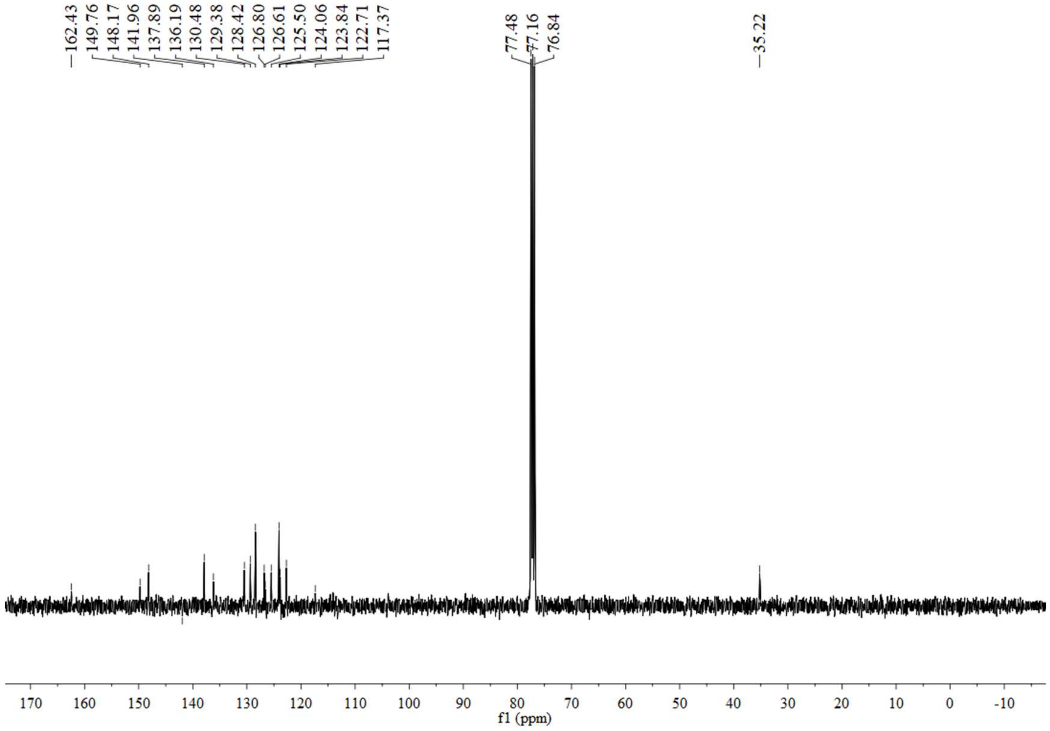

Figure S19 ^{1}H NMR Spectra of Compound of 3k

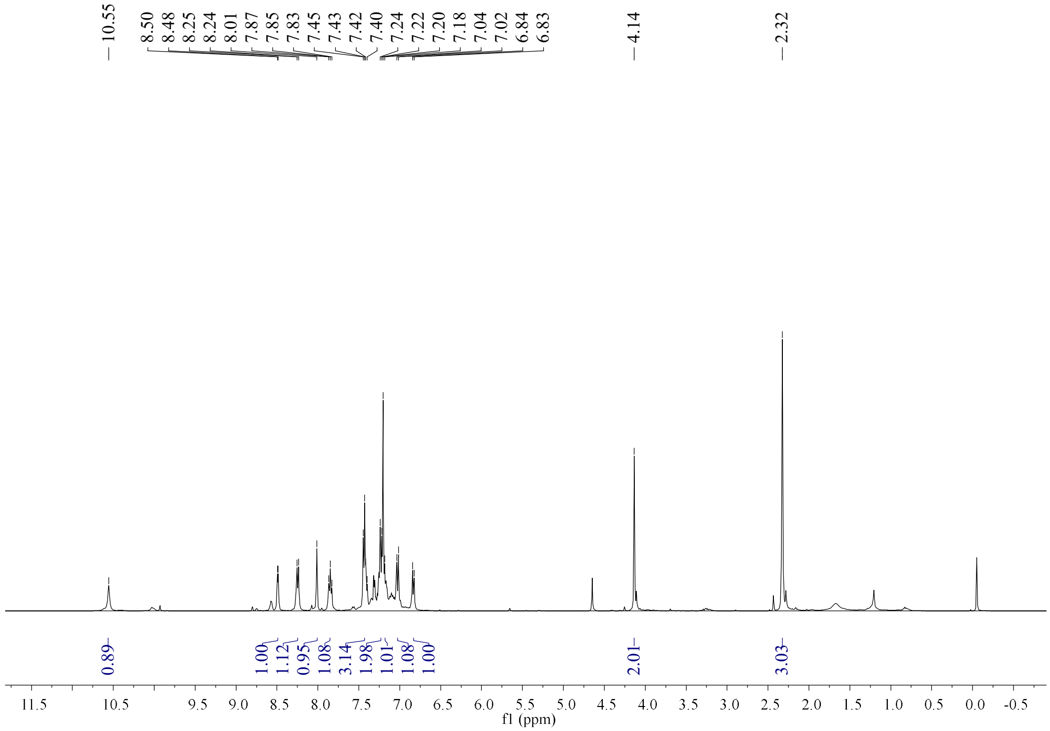

Figure S20 ^{13}C NMR Spectra of Compound of 3k

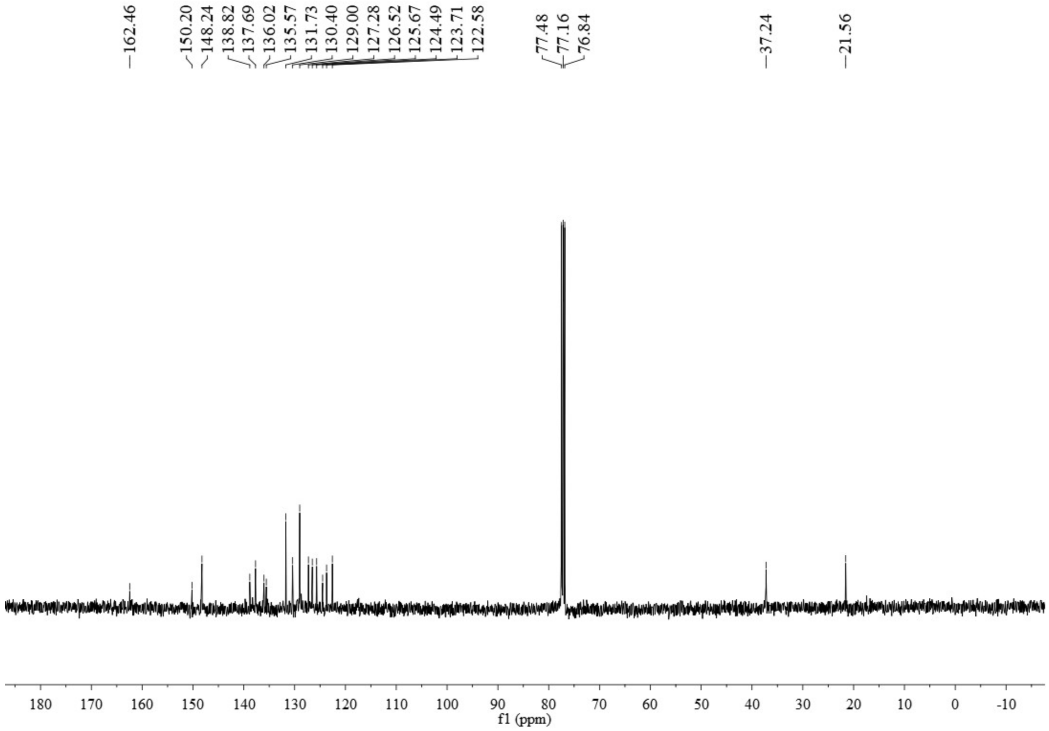

Figure S21 ^{1}H NMR Spectra of Compound of 3l

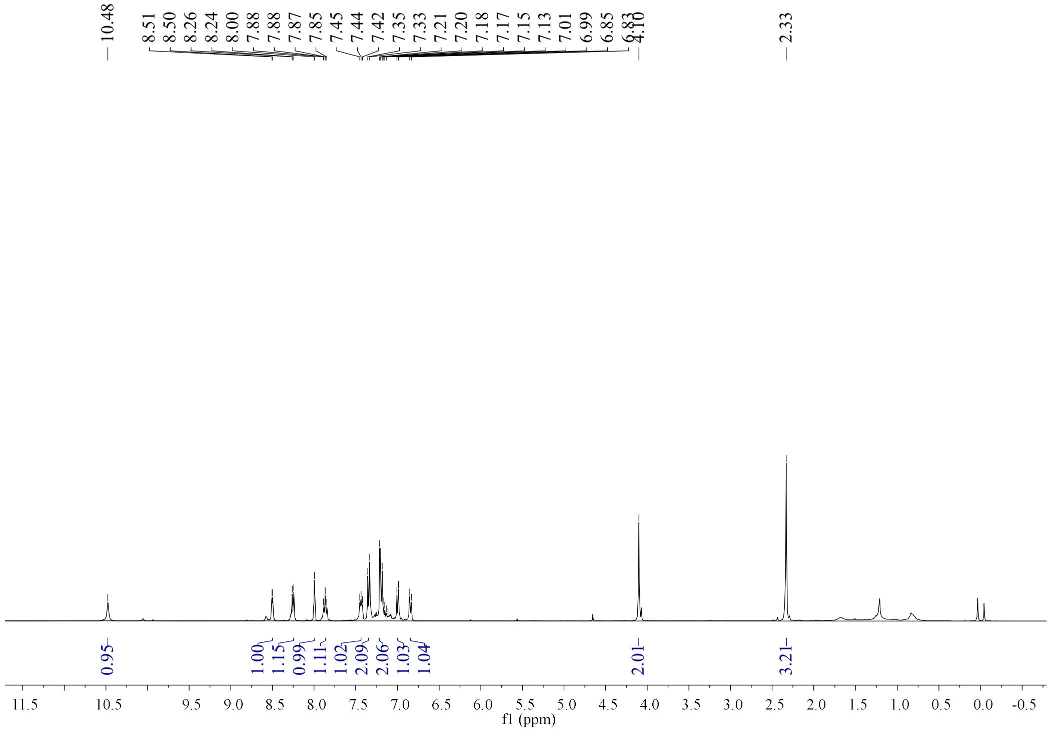

Figure S22 ^{13}C NMR Spectra of Compound of 3l

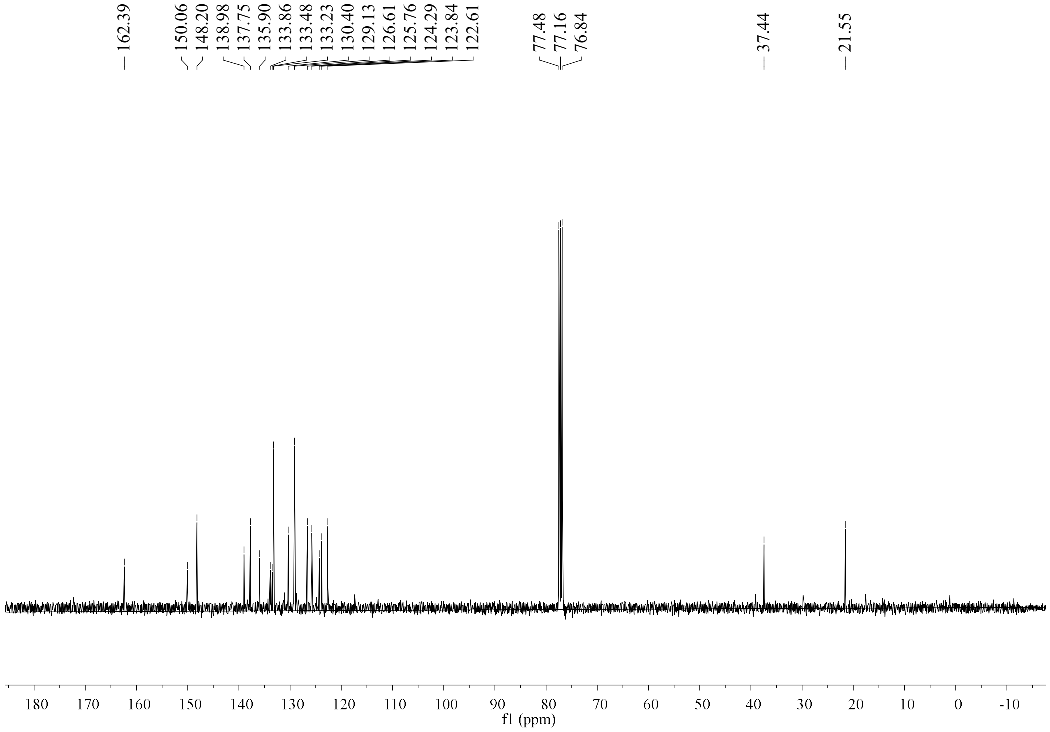

Figure S23 ¹H NMR Spectra of Compound of 3m

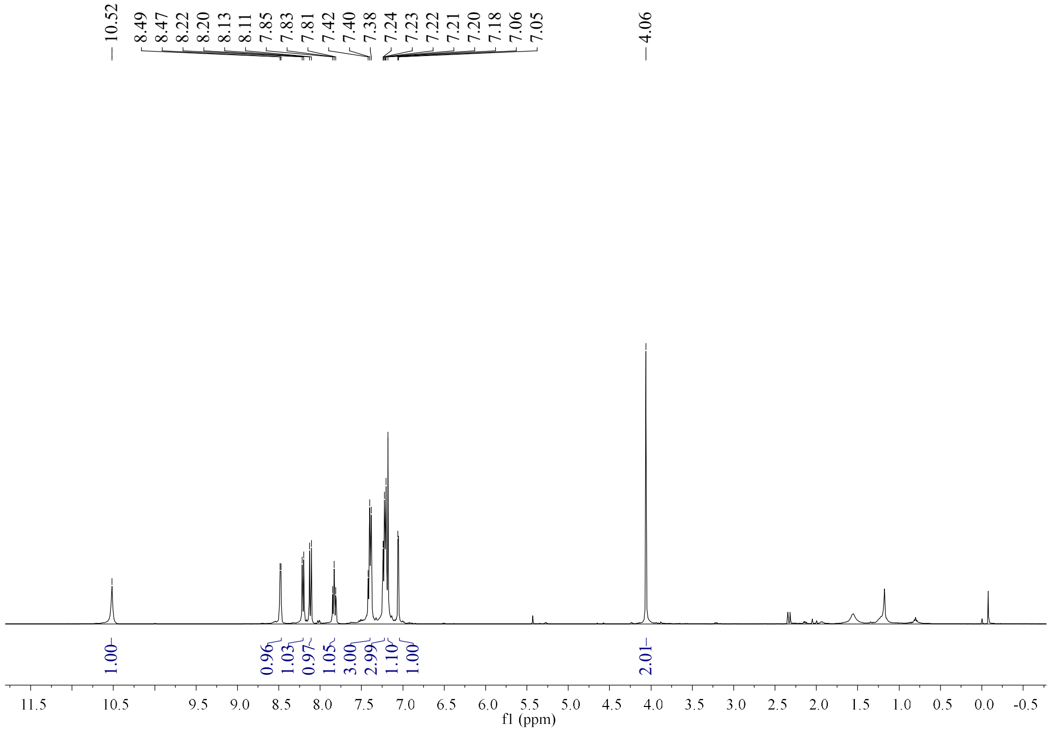

Figure S24 ¹³C NMR Spectra of Compound of 3m

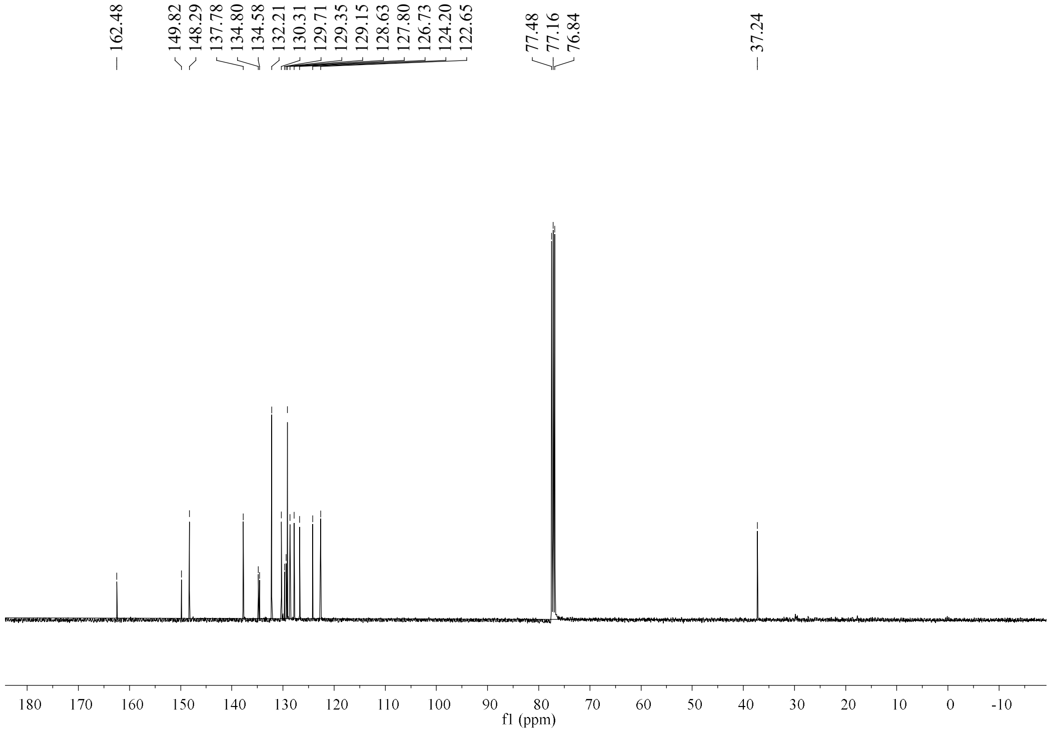

Figure S25 ^{1}H NMR Spectra of Compound of 3n

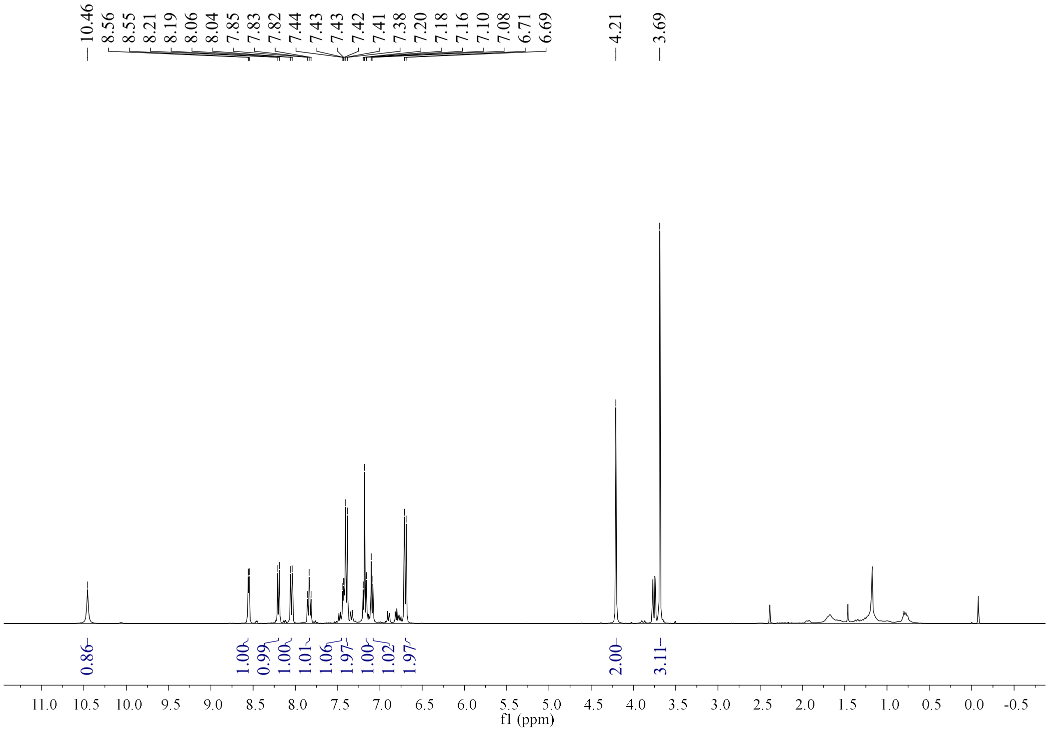

Figure S26 ^{13}C NMR Spectra of Compound of 3n

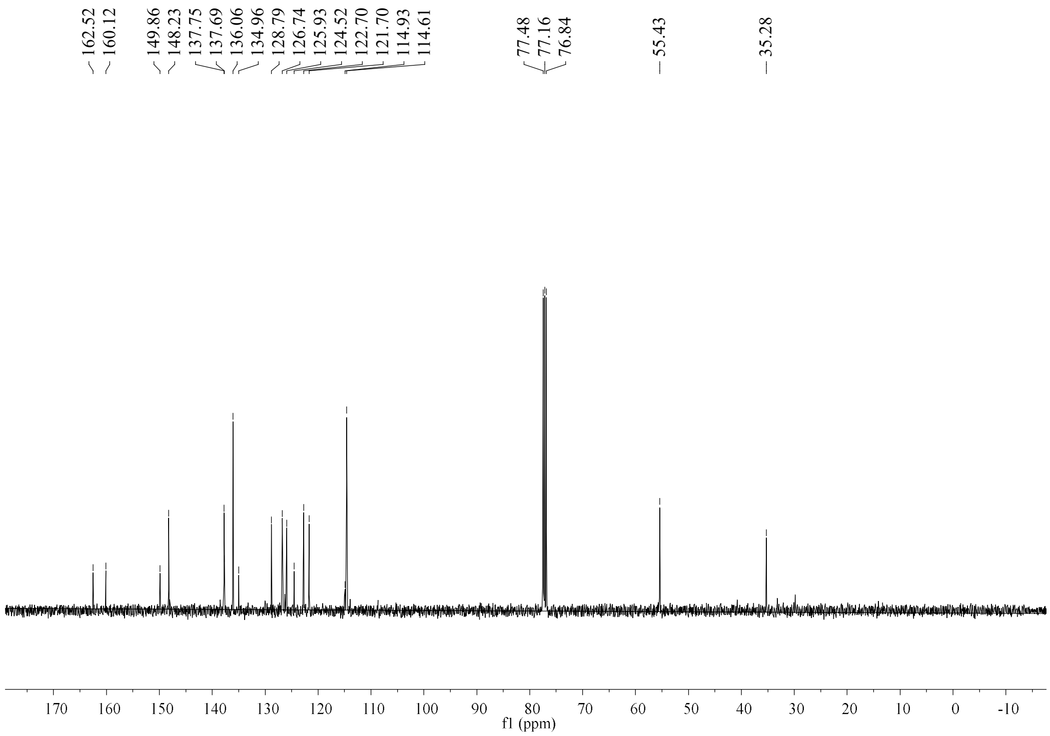

Figure S27 ^{1}H NMR Spectra of Compound of 3p

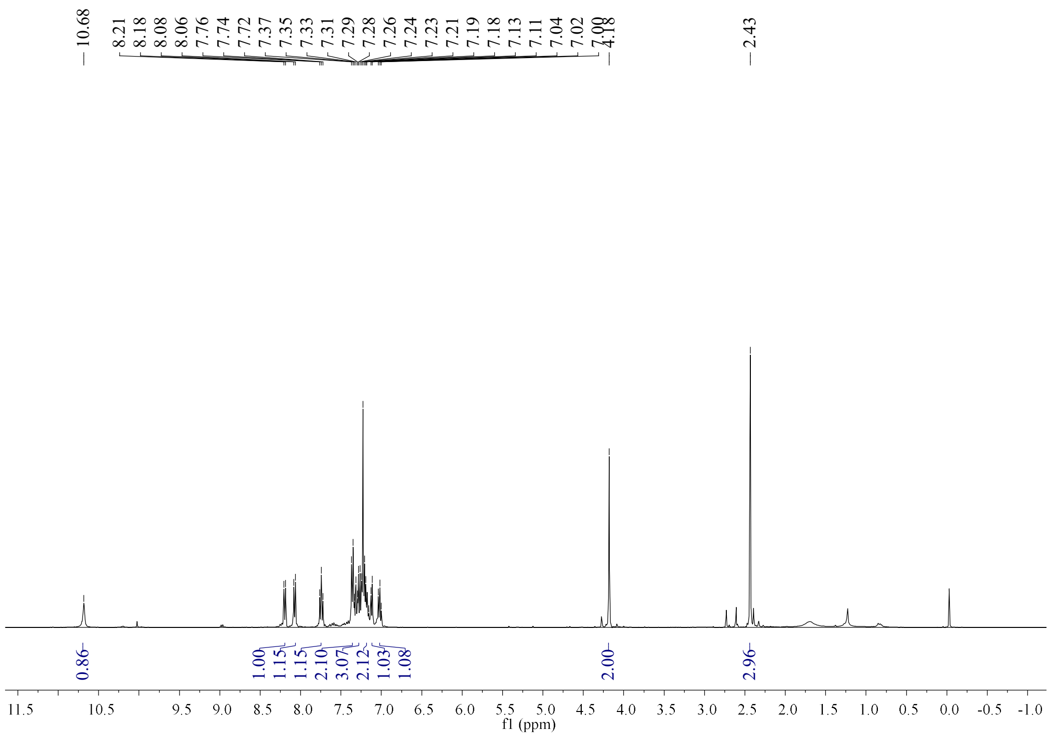

Figure S28 ^{13}C NMR Spectra of Compound of 3p

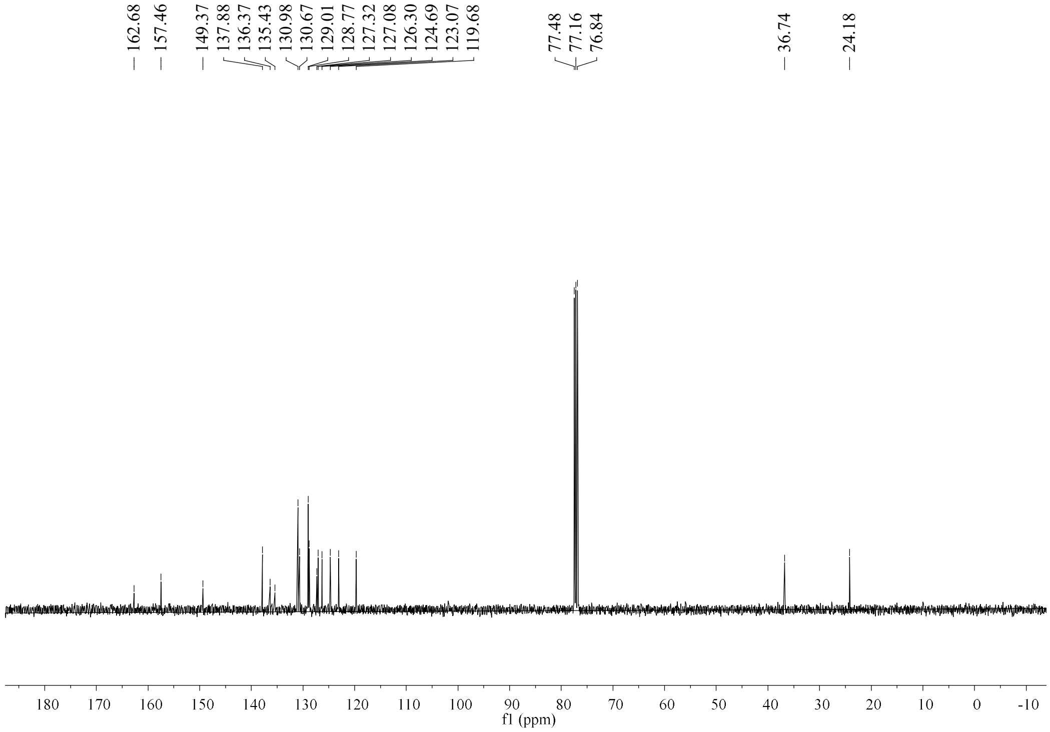

Figure S29 ^{1}H NMR Spectra of Compound of 3q

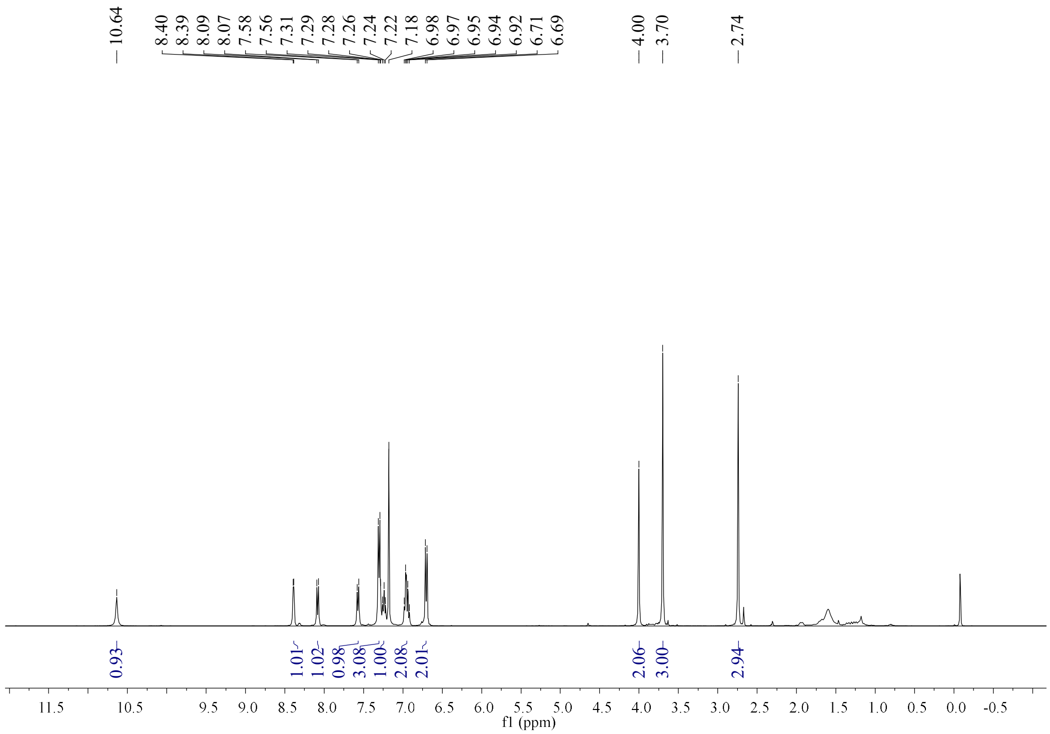

Figure S30 ^{13}C NMR Spectra of Compound of 3q

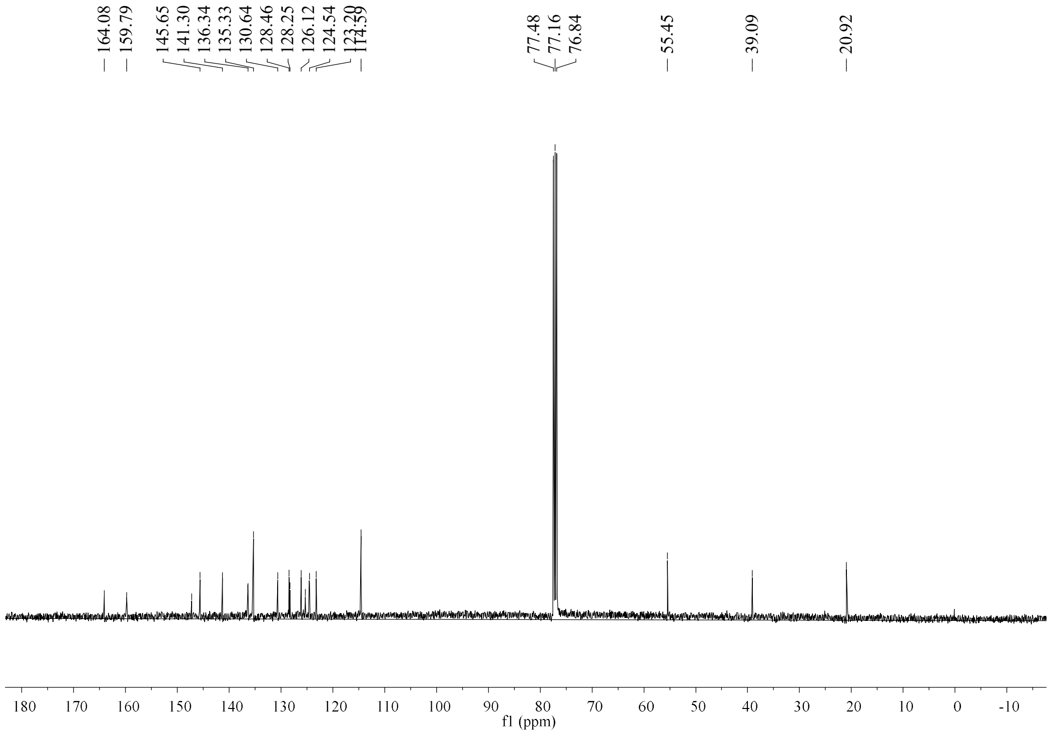

Figure S31 ^{1}H NMR Spectra of Compound of 3r

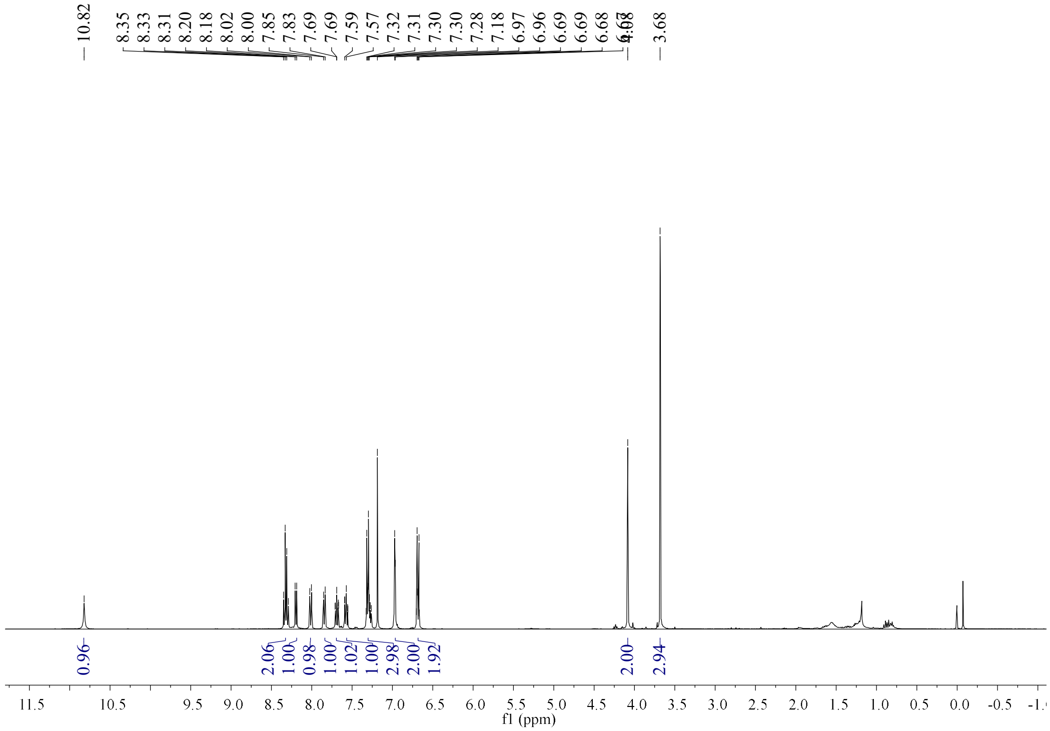

Figure S32 ^{13}C NMR Spectra of Compound of 3r

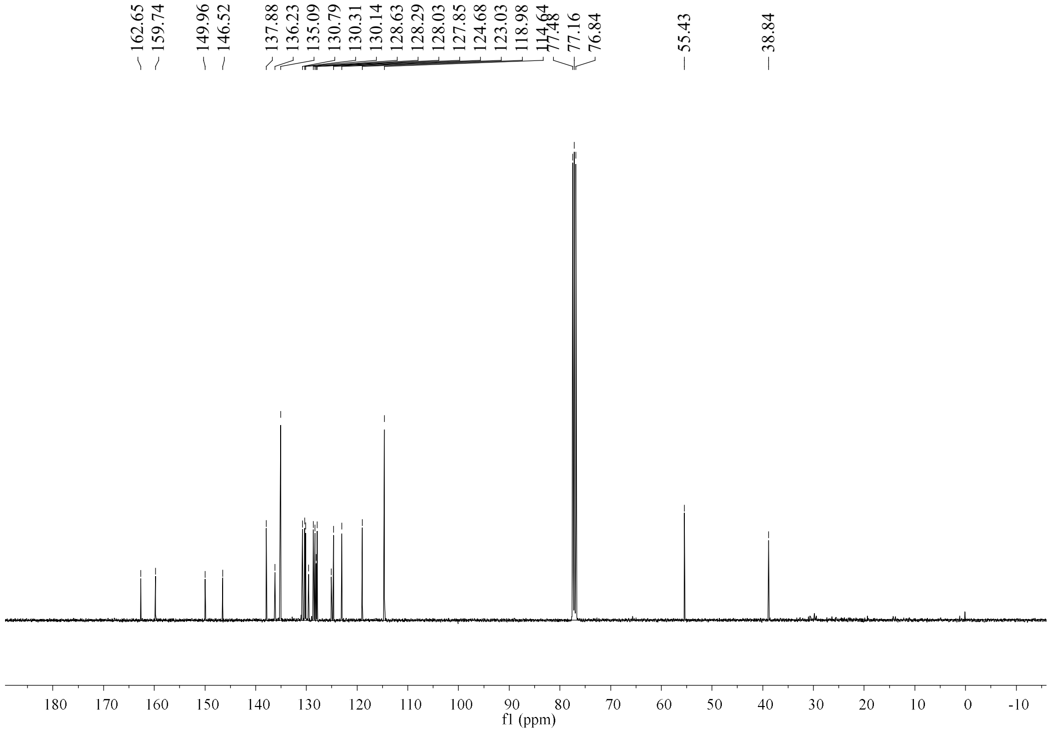

Figure S33 ^{1}H NMR Spectra of Compound of 3s

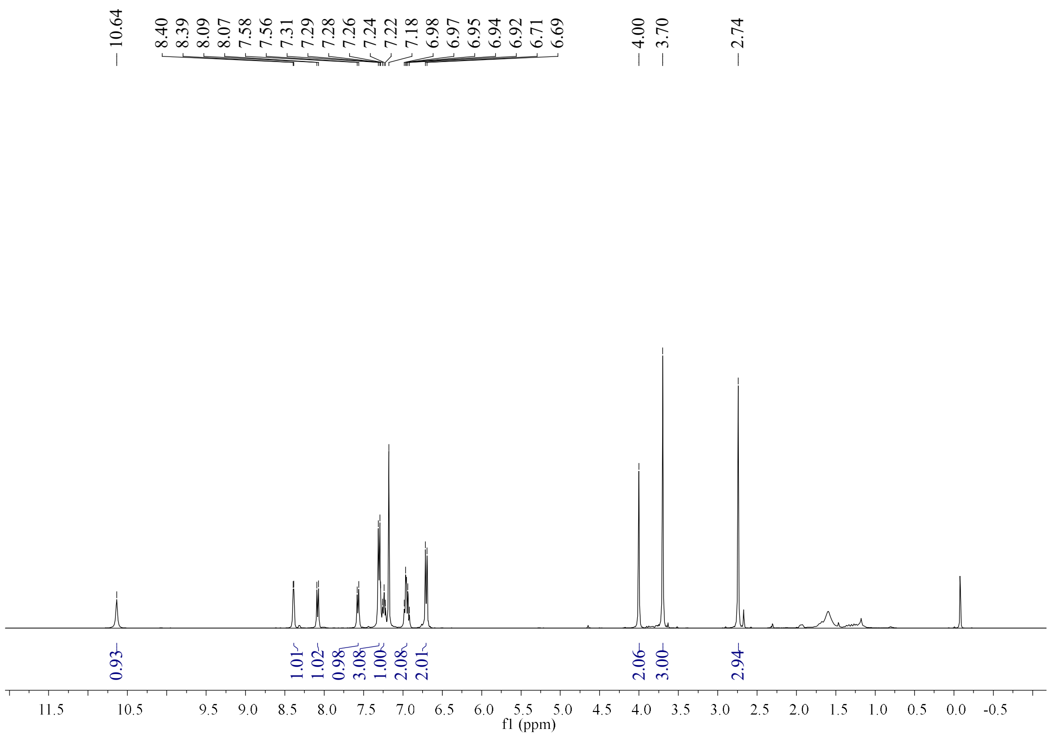

Figure S34 ^{13}C NMR Spectra of Compound of 3s

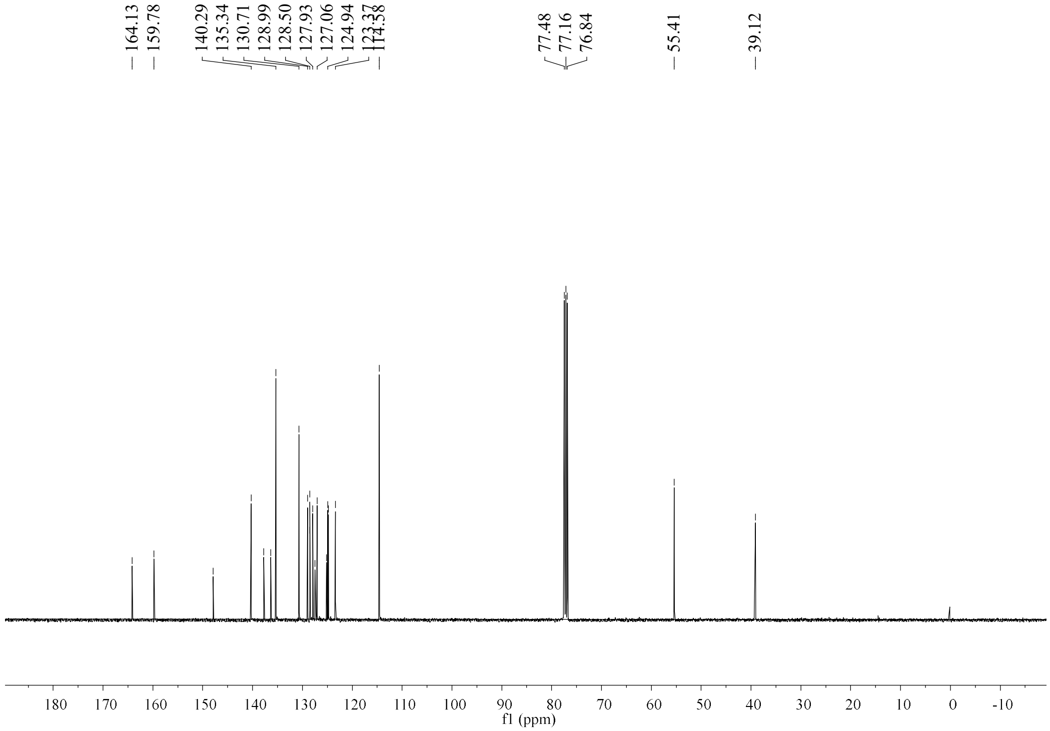

Supplement: Supplementary file 1 [file molecules-30-04462-s001.zip › molecules-3940782-supplementary.pdf]
